# Significant modulation of the hepatic proteome induced by exposure to low temperature in *Xenopus laevis*

**DOI:** 10.1242/bio.20136106

**Published:** 2013-08-23

**Authors:** Kazumichi Nagasawa, Yuta Tanizaki, Takehito Okui, Atsuko Watarai, Shinobu Ueda, Takashi Kato

**Affiliations:** 1 Department of Integrative Bioscience and Biomedical Engineering, Graduate School of Advanced Science and Engineering, Center for Advanced Life and Medical Science, Waseda University, TWIns Building, 2-2 Wakamatsu-cho, Shinjuku-ku, Tokyo 162-8480, Japan; 2 Japan Society for the Promotion of Science (JSPS), Kojimachi Business Center Building, 5-3-1 Kojimachi, Chiyoda-ku, Tokyo 102-0083, Japan; 3 Institute for Innovation Design, Comprehensive Research Organization, Waseda University, TWIns Building, 2-2 Wakamatsu-cho, Shinjuku-ku, Tokyo 162-8480, Japan; 4 Department of Biology, School of Education, Center for Advanced Life and Medical Science, Waseda University, TWIns Building, 2-2 Wakamatsu-cho, Shinjuku-ku, Tokyo 162-8480, Japan

**Keywords:** Proteomics, Pathway, Liver, Low temperature, Animal model, *Xenopus laevis*

## Abstract

The African clawed frog, *Xenopus laevis*, is an ectothermic vertebrate that can survive at low environmental temperatures. To gain insight into the molecular events induced by low body temperature, liver proteins were evaluated at the standard laboratory rearing temperature (22°C, control) and a low environmental temperature (5°C, cold exposure). Using nano-flow liquid chromatography coupled with tandem mass spectrometry, we identified 58 proteins that differed in abundance. A subsequent Gene Ontology analysis revealed that the tyrosine and phenylalanine catabolic processes were modulated by cold exposure, which resulted in decreases in hepatic tyrosine and phenylalanine, respectively. Similarly, levels of pyruvate kinase and enolase, which are involved in glycolysis and glycogen synthesis, were also decreased, whereas levels of glycogen phosphorylase, which participates in glycogenolysis, were increased. Therefore, we measured metabolites in the respective pathways and found that levels of hepatic glycogen and glucose were decreased. Although the liver was under oxidative stress because of iron accumulation caused by hepatic erythrocyte destruction, the hepatic NADPH/NADP ratio was not changed. Thus, glycogen is probably utilized mainly for NADPH supply rather than for energy or glucose production. In conclusion, *X. laevis* responds to low body temperature by modulating its hepatic proteome, which results in altered carbohydrate metabolism.

## Introduction

In order to survive, organisms respond to environmental changes by altering their expression patterns of genes and proteins. One of the most important environmental factors is temperature, which governs the distribution, behaviour, and physiological response of organisms. When the environmental temperature falls, endothermic vertebrates, i.e. mammals and birds, maintain their core body temperature within a narrow range by a thermoregulatory system. Their bodies undergo numerous physiological changes in response to cold stress to maintain their temperature (Sonna et al., 2002; Silva, 2006). A cold environment leads to hypothermia – abnormally low body temperatures. Apart from species that hibernate, most endotherms are unable to survive at low body temperatures. In contrast to endotherms, the core body temperature of ectothermic vertebrates, including most fish, amphibians, and reptiles, as well as most invertebrates, becomes low (Salt, 1949). The low body temperature conditions modulate their physiological functioning directly or indirectly due to low environmental temperature. How and why mammalian hibernators and ectothermic vertebrates survive in low temperatures have long been a scientific challenge and an important line of inquiry for biologists. The cellular and physiological responses to low body temperature have been investigated. The key response is the downregulation of the cellular metabolic rate to new hypometabolic steady states in a way that balances the ATP demand and ATP supply pathways (Hochachka, 1986; Boutilier, 2001).

Amphibians have been used as physiological models to study the responses to environmental stresses (Burggren and Warburton, 2007; Hopkins, 2007). Cold exposure depresses their metabolic rates and has effects on mitochondrial bioenergetics (Boutilier et al., 1997; Trzcionka et al., 2008). The African clawed frog, *Xenopus laevis* (Daudin), has served as a crucial vertebrate model for biological research. Since the wild habitat distribution of *X. laevis* covers a wide geographical range (Tinsley et al., 1996; Tinsley and McCoid, 1996), this species is capable of tolerating a range of environmental conditions. Therefore, we considered that *X. laevis* exposed to low temperatures would be a good hypothermic model to investigate responses to environmental temperature.

To understand the physiological responses to low-temperature stress, global analyses of the genes and proteins responding to an environmental stimulus should enable a deciphering of the underlying physiological pathways (Cossins et al., 2006; Gracey, 2007). Although a number of previous studies have compared the expression levels of mRNAs and proteins in cells and tissues, their correlation has been controversial (Anderson and Seilhamer, 1997; Ideker et al., 2001; Chen et al., 2002; Griffin et al., 2002; Mehra et al., 2003; de Sousa Abreu et al., 2009; Maier et al., 2009; Schwanhäusser et al., 2011; Ghazalpour et al., 2011). Considering that physiological events are determined by protein-driven processes, the proteome should provide key information to understand molecular responses. Proteomic studies have investigated the following associated physiological responses in *X. laevis*: the biosynthetic and secretory processes in neuroendocrine cells (van Herp et al., 2008), white/black-background adaptation (Devreese et al., 2010), response to chemical exposure (Gillardin et al., 2009; Serrano et al., 2010), photoreceptor outer segment assembly (Wang et al., 2009), and limb regeneration (King et al., 2009). DNA microarrays for *X. laevis* studies are currently commercially available from some companies (GeneChip *Xenopus laevis* Genome Array from Affymetrix, Inc. and *Xenopus* Gene Expression Microarray from Agilent Technologies, Inc.). However, the proteomic approach in *X. laevis* has been limited due to lack of availability of complete genomic information. The draft genome sequence assembly of the Western clawed frog, *Xenopus* (*Silurana*) *tropicalis*, was recently reported (Hellsten et al., 2010). Additionally, concerted efforts are currently underway in the United States, Japan, and elsewhere to carry out *X. laevis* genome sequencing [Xenopus Community White Paper 2011; Xenbase: *Xenopus laevis* and *Xenopus tropicalis* biology and genomics resource (http://www.xenbase.org/common)]. In the near future, improvement in the accuracy of the *Xenopus* genome sequence will be achieved to facilitate proteomic approaches.

We previously investigated the haematopoietic response to low temperature in *X. laevis* because haematopoiesis is one of the most important physiological functions. After 24 hours of cold exposure (5°C), *X. laevis* displays anaemia associated with hepatic erythrocyte destruction and hepatic iron accumulation as a result of heme degradation (Maekawa et al., 2012). The anaemia is prolonged during cold exposure concomitantly with hepatic confinement of newly produced erythrocytes (Maekawa et al., 2012). Generally, the liver plays a central role in metabolic homeostasis and is a major site for the synthesis, metabolism, storage, and redistribution of carbohydrates, proteins, and lipids (Bechmann et al., 2012). The liver also plays an important role in energy metabolism and the large change in metabolic rate caused by cold exposure. In *X. laevis*, in contrast to mammals and terrestrial frogs, the liver also plays a central role in the production and destruction of erythrocytes (Chegini et al., 1979; Nogawa-Kosaka et al., 2010; Nogawa-Kosaka et al., 2011; Maekawa et al., 2012; Okui et al., 2013). Therefore, after 24 hours of cold exposure, various physiological responses may occur in the liver. It is important to investigate these initial responses to cold-exposure-induced low body temperature.

In the present study, we used a proteomics approach to profile the liver proteome in *X. laevis* after exposure to low temperature, because proteomics studies on the liver at low body temperature, such as gilthead sea breams exposed to the cold (Ibarz et al., 2010), mammalian hibernators during entrance into hibernation (Epperson et al., 2004; Shao et al., 2010; Epperson et al., 2010; Rose et al., 2011), rat induced hypothermia (Oda et al., 2012), and freeze-tolerant wood frogs during winter (Kiss et al., 2011) were recently reported. These studies enable us to carry out cross-species comparisons of liver proteome changes. We applied a label-free quantification method using nano-flow liquid chromatography coupled with tandem mass spectrometry (nanoLC–MS/MS) to assess *X. laevis* liver proteins that differ in abundance between standard laboratory rearing temperature (22°C, control condition) and low environmental temperature (5°C, cold exposure). The purpose of this study was to gain an insight into the initial physiological response to cold-exposure-induced low body temperature.

## Materials and Methods

### Animals

Wild-type *X. laevis* (mass 30–40 g) frogs were purchased from Kazuo Ouchi (Misato, Saitama, Japan) and housed in plastic tanks at the standard laboratory rearing temperature (22°C) with constantly running water. This condition was defined as the control condition. For low-temperature exposure, plastic tanks containing *X. laevis* (one frog per tank containing 1 l of water at 22°C) were transferred to an incubator (Bio Multi incubator; NK Systems, Osaka, Japan) set at 5°C and allowed to cool. All experiments were conducted according to the Regulations for Animal Experimentation at Waseda University.

### Haematological analysis

The haematological value of peripheral blood, including blood cell counts, haemoglobin, and haematocrit values were obtained as previously reported (Aizawa et al., 2005; Nogawa-Kosaka et al., 2010; Nogawa-Kosaka et al., 2011; Maekawa et al., 2012).

### Liver tissue collection

At 24 hours after cold exposure, *X. laevis* were quickly killed by beheading. The livers from each of the control and cold-exposure groups (*n* = 3 each) were cut into smaller pieces and flushed with Tris-buffered saline (20 mmol l^−1^ Tris-HCl pH 7.5, 100 mmol l^−1^ NaCl) containing 1 mmol l^−1^ ethylenediaminetetraacetic acid (TBSE) to remove the excess blood, and then directly frozen in liquid nitrogen and stored at −80°C for protein analyses.

### Extraction of hepatic proteins

A piece of the liver was homogenized in TBSE (0.5 ml per 100 mg of tissue) using a bead beater-type homogenizer (Beads Crusher μT-12; TAITEC Co., Ltd., Saitama, Japan). The liver homogenates were centrifuged at 1,500×*g* for 5 minutes at 4°C to remove cell debris and the supernatants were further centrifuged at 15,000×*g* for 20 minutes at 4°C to remove insoluble proteins. The supernatants containing soluble protein were collected and stored at −80°C until use. Protein concentration was determined using the Bradford assay reagent.

### Protein digestion

Three protein extracts from each individual were mixed in equal amounts (Fig. 2). The mixed extract containing 50 µg of protein was dissolved in 0.5 mol l^−1^ Tris-HCl (pH 8.5) containing 8 mol l^−1^ urea, 2.5 mmol l^−1^ ethylenediaminetetraacetic acid, and 10 mmol l^−1^ dithiothreitol, and incubated for 1.5 hours at 37°C. Iodoacetamide was then added at a concentration of 50 mmol l^−1^ to alkylate the reduced thiol groups. After incubation for 30 minutes at room temperature in the dark, the mixture was diluted with 50 mmol l^−1^ ammonium bicarbonate buffer at a final concentration of 1 mol l^−1^ urea. For digestion, sequencing grade modified trypsin (Promega KK., Tokyo, Japan) was added to the protein solution at 1:50 (trypsin:protein) and the mixture was incubated for 19 hours at 37°C. Formic acid was added at a concentration of 0.1% to stop the reaction. The final solution was cleaned up with MonoTip C18 tips (GL Sciences Inc., Tokyo, Japan) and the eluent was evaporated in a vacuum centrifuge. The powdered peptides were redissolved in 50 µl of 2% acetonitrile in water containing 0.02% formic acid for nanoLC–MS/MS analysis.

### nanoLC–MS/MS analysis

We used Nano Frontier eLD system (Hitachi High-Technologies Corporation, Tokyo, Japan). The analytical column was a packed nano-capillary column (NTCC-360/75-3; Nikkyo Technos Co. Ltd., Tokyo, Japan). We also used a monolithic trap column (Monolith Trap C18-50-15, Hitachi High-technologies Corporation, Tokyo, Japan). The LC conditions were as follows. The flow rate of the nanoflow pump was set at 200 nl min^−1^. Solvent A was 2% aqueous acetonitrile containing 0.1% formic acid, and solvent B was 98% aqueous acetonitrile containing 0.1% formic acid. The composition of solvent B was linearly increased from 2% at 0 minutes to 35% at 150 minutes, maintained at 100% until 165 minutes, and then returned to the initial condition of 2%. The MS/MS conditions were as follows: ESI voltage, +1.6 kV; curtain (nitrogen) gas flow rate, 0.6 l min^−1^; precursor mass scan range, m/z 100–2000; scan time, 20 msec; fragment mass scan range, m/z 50–2000. The 2 µl of peptide solution (equivalent to 2 µg of protein) were analysed in triplicate runs (Fig. 2).

### Data processing

Xome (Mitsui Knowledge Industry Co., Ltd., Tokyo, Japan) software was used for generating the peak list and identification of proteins (Honmyo, 2007). For the identification of proteins, we performed peptide mass fingerprint against the NCBInr database [NCBInr 20130303 (23463169 sequences; 8064228071 residues); National Center for Biotechnology Information, http://www.ncbi.nlm.nih.gov] using the Mascot search engine version 2.1 (Matrix Science, London, UK). The Mascot MS/MS ion search conditions were as follows: taxonomy filter, *X. laevis* (African clawed frog) (17403 sequences); enzyme, trypsin; maximum missed cleavages, 1; fixed modifications, carbamidomethyl (C); variable modifications, oxidation (M); peptide mass tolerance, 0.3 Da; MS/MS ion mass tolerance, 0.3 Da; charge states, +1, +2, and +3; mass values, monoisotopic; instrument type, ESI-TRAP. The threshold score to achieve *P*<0.05 was set by the Mascot algorithm. Because the nanoLC–MS/MS was performed in triplicate, the triplicate data were processed separately (Fig. 2). As a result, the false discovery rate (FDRs) was less than 0.05 in all Mascot searches, which indicates that the significance threshold (*P*<0.05) was applicable (Fig. 3A). The complete results are listed in supplementary material Table S1. Only an identification observed in at least two of the three replicates was taken to be valid (Fig. 3B). The Xome programme identified 145 *X. laevis* proteins, 113 of which had a human homologue in the NCBI HomoloGene database (http://www.ncbi.nlm.nih.gov/homologene). The remaining 32 proteins were manually annotated by alignment with the NCBI Reference Sequence (RefSeq) database using the BLASTp programme (NCBI Basic Local Alignment Search Tool, http://blast.ncbi.nlm.nih.gov/Blast.cgi).

To screen proteins differentially expressed between the control and cold-exposure groups, fold changes in protein abundance were calculated using the ‘non-label quantitation’ function of Mass Navigator v1.2 (Mitsui Knowledge Industry Co., Ltd., Tokyo, Japan). The procedure for the calculation of protein fold changes is described in Table 1. The complete results are listed in supplementary material Table S2.

**Table 1.**
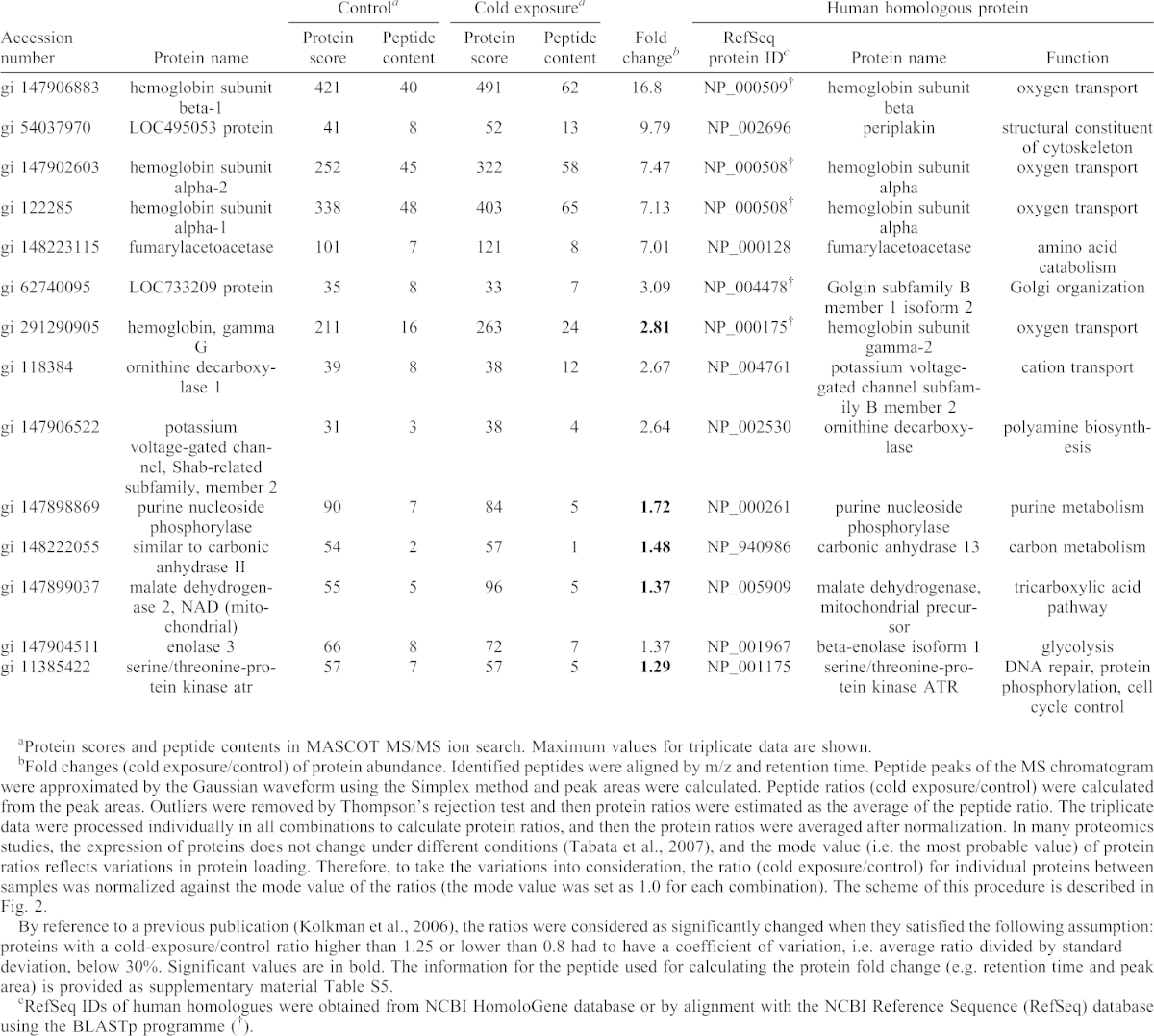
Upregulated proteins in cold-exposed *X. laevis* liver (group 1).

### Gene ontology and pathway analysis

To identify significantly represented biological themes and functional groups in the protein list, gene ontology (GO) and pathway analysis were performed using the Database for Annotation, Visualization and Integrated Discovery (DAVID) v6.7 program (http://david.abcc.ncifcrf.gov) (Huang et al., 2009a; Huang et al., 2009b). The GO analysis was used to identify enriched biological themes using GO terms defined and provided as official terms by the Gene Ontology Consortium (http://www.geneontology.org) (Dennis et al., 2003). The ‘biological process’ subontology of GO (GO:BP) refers to a biological objective to which the protein contributes and is widely used to evaluate sets of relationships between proteins. The pathway analysis was used to identify candidate proteins involved in pathways from the Kyoto Encyclopaedia of Genes and Genomes (KEGG) pathway database (http://www.genome.jp/kegg/pathway.html). The analysis conditions were as follows: when the list of identified proteins in the control and cold-exposure conditions was used as the input data, the DAVID default population background (corresponding genome-wide genes with at least one annotation in the analysing categories) was used; when the differentially expressed protein list was used, the total protein list from this study (145 proteins) was inputted and used as a customized population background. We used EASE scores, which modified Fisher's exact test *P* values to evaluate the significance of enrichment (Hosack et al., 2003), and Benjamini and Hochberg FDR procedures for multiple testing corrections (Benjamini and Hochberg, 1995). Only statistically enriched GO terms and pathways (Benjamini and Hochberg FDR-corrected *P*<0.01) with at least two proteins were selected. To determine the hierarchical structure of the selected GO terms, we used QuickGO (http://www.ebi.ac.uk/QuickGO).

### Quantification of free amino acids

Free amino acids were quantified by EZ:faast GC/FID Free (Physiological) Amino Acid Kit (Phenomenex, Inc., CA, USA) by using gas chromatography (GC) equipped with a flame ionization detector (FID) (GC-2014; Shimadzu Corporation, Kyoto, Japan). The protein extracts from *X. laevos* liver (equivalent to 0.5 mg protein) were subjected to pretreatment for GC/FID analysis according to the manufacturer's protocol. Chromatographic conditions were as follows: initial oven temperature of 80°C with 20°C min^−1^ ramps to the final temperature of 320°C. Inlet temperature of 280°C under constant helium flow of 50 cm sec^−1^ and the detector temperature of 320°C is used for the analysis of derivatized amino acids.

### Quantification of glycogen in the liver

Liver glycogen was isolated by precipitation from ice-cold 5% trichloroacetic acid extracts using ice-cold 70% ethanol as previously described (Graff and Allen, 1963). The precipitate was dissolved in water, hydrolyzed to glucose for 2 hours at 100°C in 2 N sulphuric acid, and neutralized using 2 N sodium hydrate (Sahyun, 1931). The glucose was measured using the glucose oxidase method (Glucose CII Test-Wako; Wako Pure Chemical Industries, Ltd., Osaka, Japan). The absorbance at 505 nm was measured using a microplate reader (POWERSCAN HT; DS Pharma Biomedical Co., Ltd., Osaka, Japan), and a factor of 0.927 was used to convert the values obtained from glucose into values for anhydrous glycogen.

### Quantification of glucose, glycerol, NADP and NADPH

Glucose in the plasma and liver extracts was measured directly using the Glucose CII Test from Wako. Glycerol in the plasma was measured directly by using the Glycerol Colorimetric Assay Kit (Cayman Chemical Company, MI, USA). Liver extracts for the glycerol assay were prepared as previously described (Driedzic et al., 2006). A piece of frozen liver was homogenized in nine volumes of 10% perchloric acid, and the homogenate was centrifuged at 15,000×*g*. The supernatant was neutralized using potassium hydrate and assayed as described above. The absorbance at 540 nm was measured. NADP and NADPH in the liver were measured using a fluorimetric assay (SensoLyte NADP/NADPH Assay Kit; AnaSpec, Inc., CA, USA) according to the manufacturer's instructions. The resulting red fluorescence was monitored at excitation and emission wavelengths of 560 and 590 nm, respectively.

## Results

### Anaemia is induced by cold exposure in *X. laevis*

**Fig. 1.**
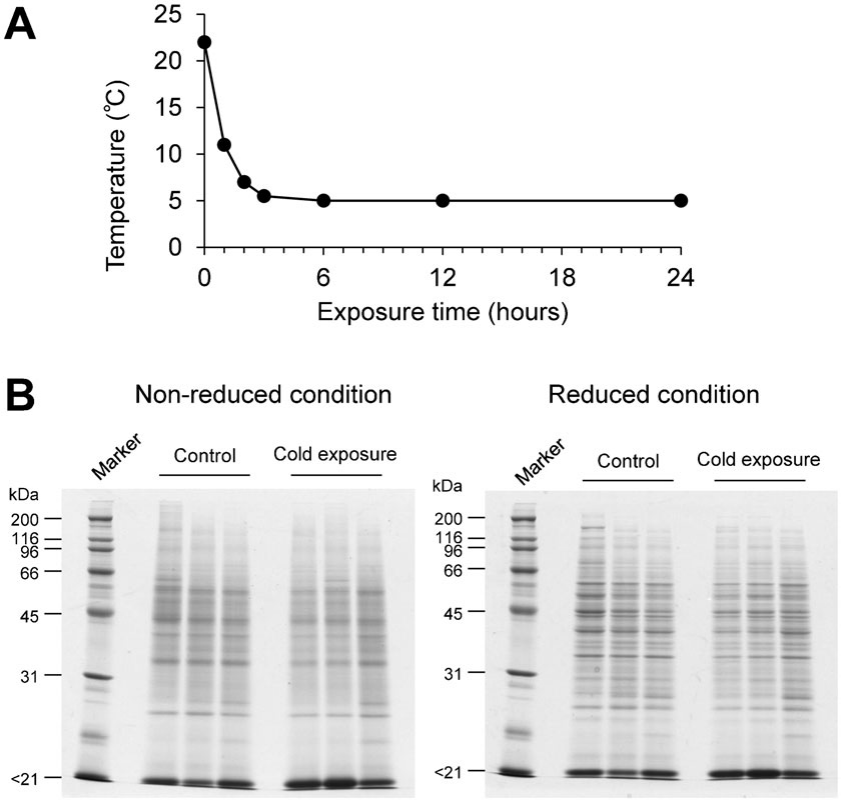
Transitions in environmental temperature and hepatic proteins. (A) Relationship between cold-exposure time and water temperature. A plastic tank containing 1 l of 22°C water was transferred to an incubator at 5°C and allowed to cool. During that time, the temperature of the water was measured. (B) Image of SDS-PAGE gels used to separate *X. laevis* liver protein samples; 10 µg of each sample from either the control or cold-exposure group (three frogs each) was separated on 12% SDS polyacrylamide gels. Protein bands were visualized by staining with Coomassie brilliant blue R-250. Reducing (right panel) and non-reducing (left panel) conditions are shown.

Over 6 hours of exposure to 5°C, the water temperature decreased to almost 5°C (Fig. 1A). At 24 hours, the erythrocyte count was approximately 70% of that in the control condition, and haemoglobin and haematocrit values were also decreased as reported previously (Maekawa et al., 2012) (data not shown). No significant changes in body or hepatic weight were observed (data not shown).

### *X. laevis* liver proteome

**Fig. 2.**
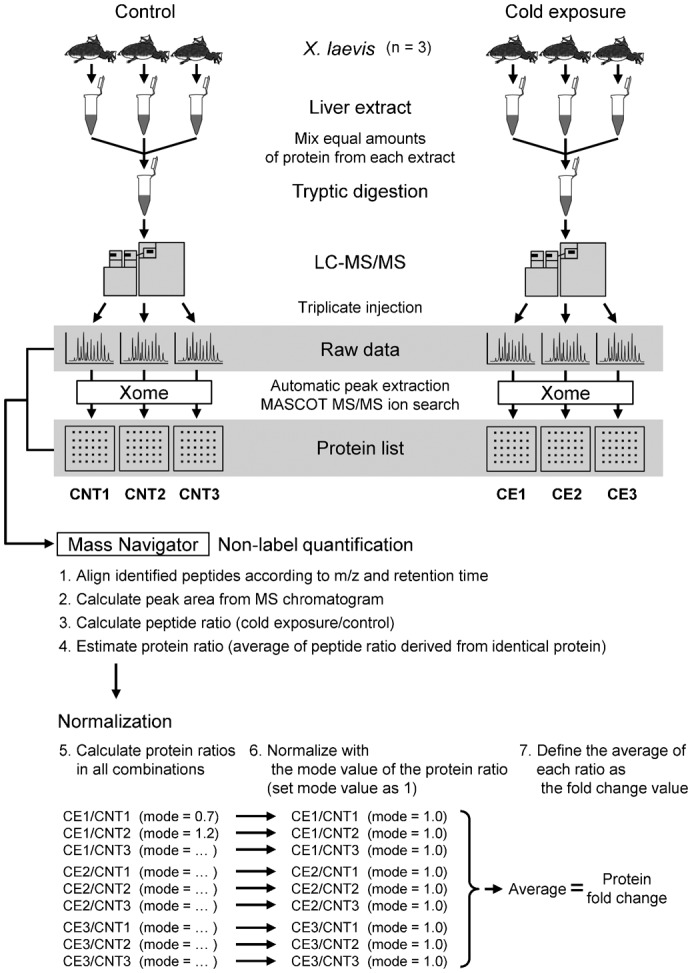
Schematic view of sample and data processing. Each test sample, i.e. a protein extract mixture derived from three frogs prepared by bead crusher, was subjected to tryptic digestion. Subsequent nanoLC–MS/MS analysis was repeated three times. Automatic peak extraction and MASCOT MS/MS ion search were performed using Xome software. The triplicate data were processed individually in all combinations to calculate the protein ratios by non-label quantification using Mass Navigator software then protein ratios were averaged after normalization. The details are explained in the Materials and Methods and in Table 1.

To survey hepatic protein profiles of *X. laevis* in the control and cold-exposure groups, we conducted a proteomics analysis using label-free nanoLC–MS/MS. Although the SDS-PAGE patterns of the Coomassie brilliant blue-stained liver extracts were similar between control and cold-exposure groups (Fig. 1B), the LC–MS/MS analysis detected differences in the protein contents. We identified 145 proteins (126 proteins from the control group and 100 proteins from the cold-exposure group), and 81 proteins overlapped between the groups (Fig. 2C). Next, we set out to find biologically relevant themes within the list of identified proteins.

**Fig. 3.**
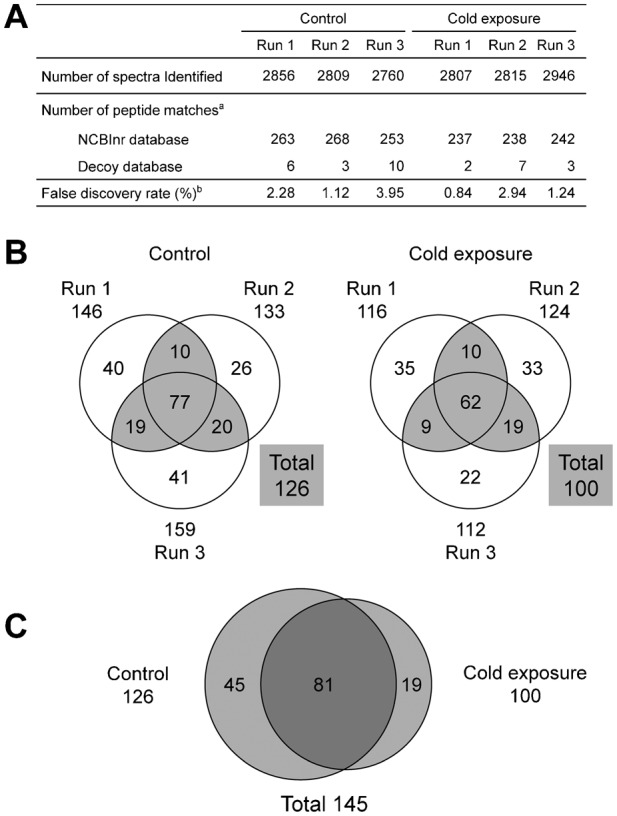
Outlines of MASCOT MS/MS ions search. (A) Summary of MASCOT peptide identification in each search. *^a^*Peptide matches above identity threshold (*P*<0.05); *^b^*FDR  =  decoy hits/NCBInr hits. (B) Venn diagrams of identified proteins in individual searches. Proteins identified in at least two of the triplicate runs are highlighted in grey. (C) Comparison of valid identified proteins differentially expressed between the control and cold-exposure conditions. Proteins identified at least twice were compared (126 proteins from the control group and 100 proteins from the cold-exposure group); 81 proteins overlapped between both groups.

**Fig. 4.**
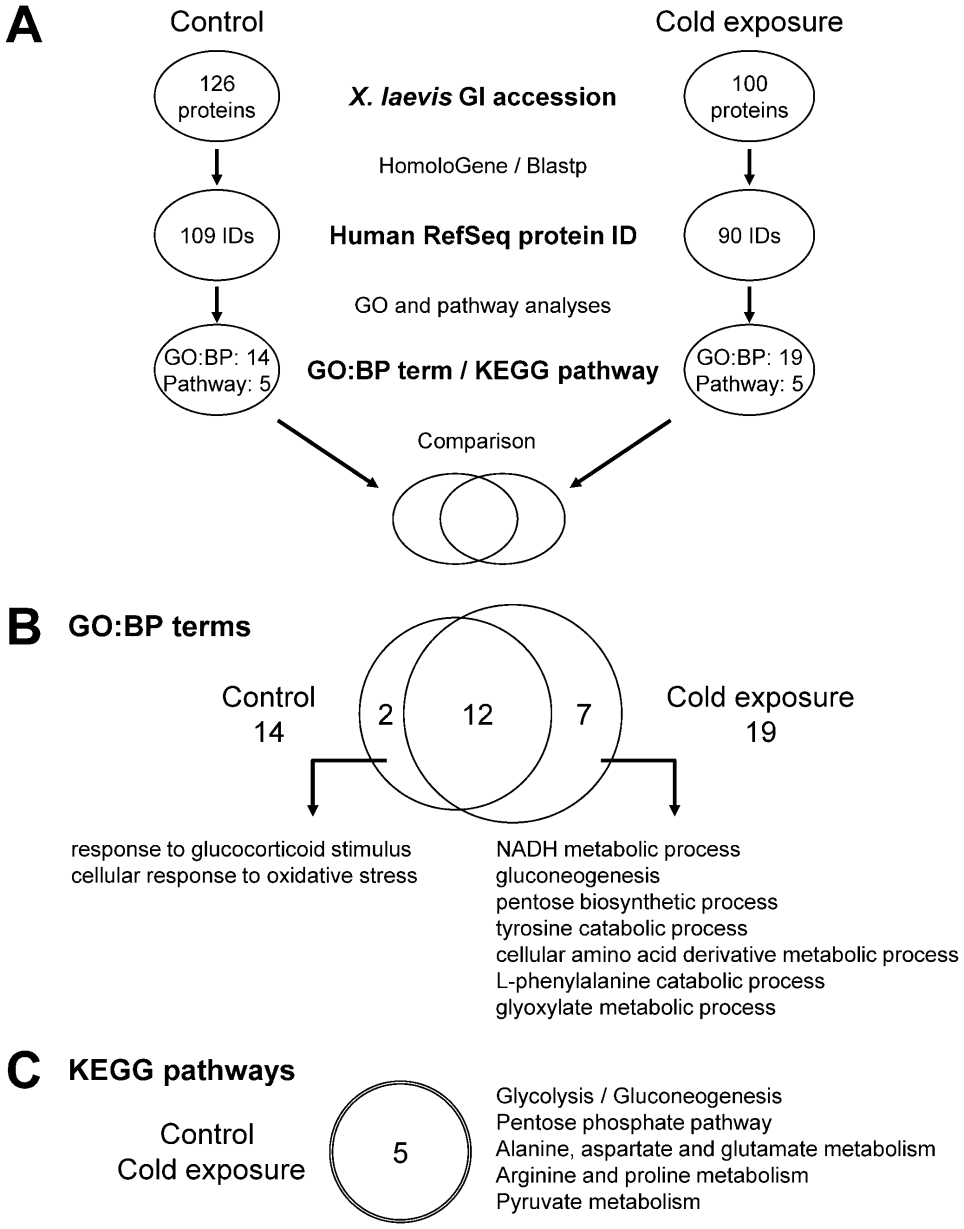
GO and pathway analyses of *X. laevis* liver proteomes. (A) Schematic view of the data processing procedure. The GI accessions of *X. laevis* proteins were converted to human RefSeq protein IDs by using NCBI HomoloGene and BLASTp, and then GO and pathway analyses were performed by using the DAVID program. FDR-corrected *P* values were defined by modified Fisher's exact test with the Benjamini and Hochberg FDR correction. The significantly identified GO:BP terms appearing deepest in the hierarchy and the significantly identified KEGG pathways are shown (FDR-corrected *P*<0.01). The details are explained in the materials and methods. (B,C) Comparison of enriched GO:BP terms (B) and KEGG pathways (C) in the list of proteins identified between the control and cold-exposure conditions.

The GO analysis identified five and seven GO:BP terms that were significantly enriched in the control and cold-exposed *X. laevis* liver proteomes, respectively. Four of these terms were common between control and cold exposure. Only one term, ‘oxygen and reactive oxygen species metabolic process (GO:0006800)’ (4 proteins, *P* = 2.87×10^−3^), was control-specific, and three terms, ‘cellular amide metabolic process (GO:0043603)’ (4 proteins, *P* = 7.17×10^−3^), ‘coenzyme metabolic process (GO:0006732)’ (6 proteins, *P* = 5.89×10^−3^), and ‘cellular amino acid metabolic process (GO:0006520)’ (9 proteins, *P* = 6.73×10^−4^), were cold-exposure-specific. In the pathway analysis, two pathways, ‘glycolysis/gluconeogenesis (xla00010)’ (control: 15 proteins, *P* = 2.62×10^−9^; cold exposure: 12 proteins, *P* = 3.63×10^−7^) and ‘pentose phosphate pathway (xla00030)’ (control: 9 proteins, *P* = 8.29×10^−7^; cold exposure: 9 proteins, *P* = 3.48×10^−7^), were commonly associated with the control and cold-exposed liver proteomes in *X. laevis*. Three pathways, ‘tyrosine metabolism (xla00350)’ (6 proteins, *P* = 1.27×10^−3^), ‘pyruvate metabolism (xla00620)’ (6 proteins, *P* = 9.86×10^−3^), and ‘arginine and proline metabolism (xla00330)’ (6 proteins, *P* = 9.48×10^−3^), were cold-exposure-specific. Minimal difference was observed between the control and cold-exposure conditions, possibly because of the small number of *X. laevis* proteins annotated with GO and KEGG pathways. We then converted the identified proteins to their homologous human proteins for analysis (Fig. 4A) and found that 14 and 19 GO:BP terms were significantly enriched in the control and cold-exposed *X. laevis* liver proteomes, respectively (Fig. 4B). Twelve of these terms were shared between control and cold exposure, and two and seven terms were control- and cold-exposure-specific, respectively (Fig. 4B; supplementary material Table S3). Five pathways were commonly associated with both control and cold-exposed *X. laevis* liver proteomes (Fig. 4C; supplementary material Table S4).

### Differential protein abundance under cold exposure

**Fig. 5.**
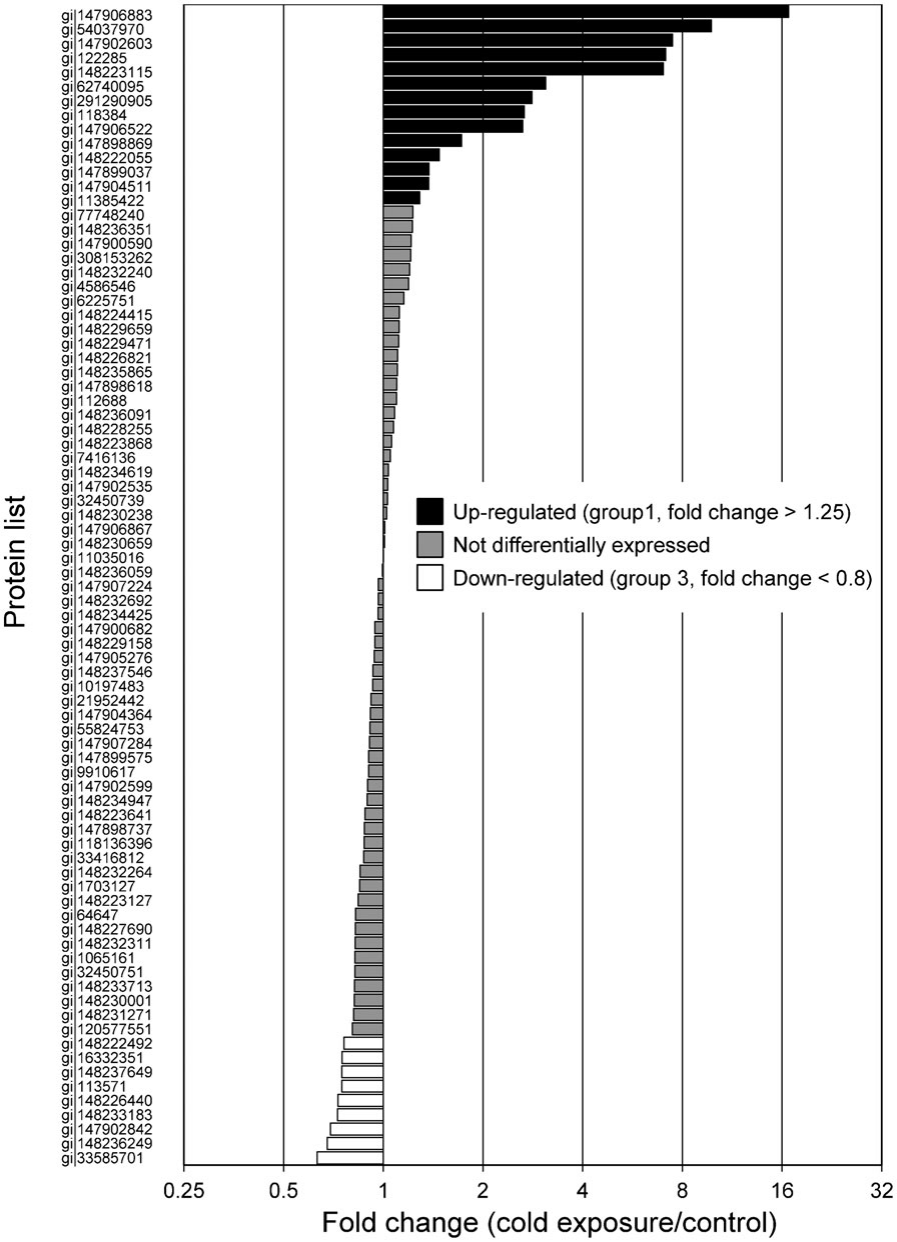
Distribution of fold change in protein abundance. The bar chart shows protein ratios between the cold-exposure and control conditions for all 81 relatively quantified proteins.

To identify differentially expressed proteins associated with cold exposure, non-label quantification was performed (Fig. 5). The proteins found to be differentially expressed were categorized into four groups. Fourteen proteins were upregulated by cold exposure (fold change >1.25) (group 1; Table 1), and 13 proteins were detected only in the cold-exposure condition (i.e. considered to be newly induced) (group 2; Table 2). Nine proteins were downregulated under cold exposure (fold change <0.8) (group 3; Table 3), and the levels of 22 proteins were below the detection limit (i.e. detected only in the control condition) (group 4; Table 4). Generally, proteins in groups 1 and 2 (total, 27) can all be viewed as upregulated and those in groups 3 and 4 (total, 31) can be viewed as downregulated under cold exposure. We then analysed the GO enrichment of these upregulated and downregulated proteins by using the DAVID program with *X. laevis* proteins and candidate human homologues. Neither enriched GO:BP terms nor associated KEGG pathways were identified, presumably because of the small number of proteins.

**Table 2.**
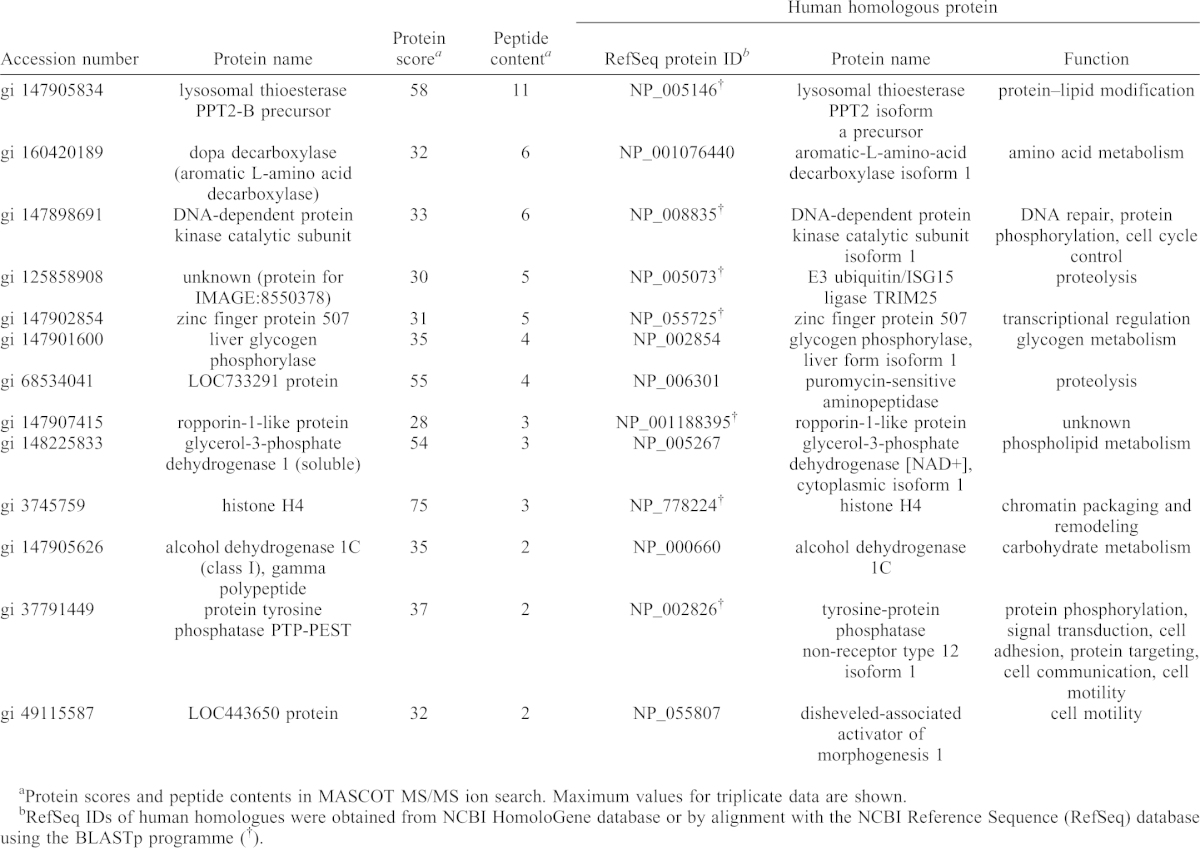
Newly induced proteins in cold-exposed *X. laevis* liver (group 2).

**Table 3.**
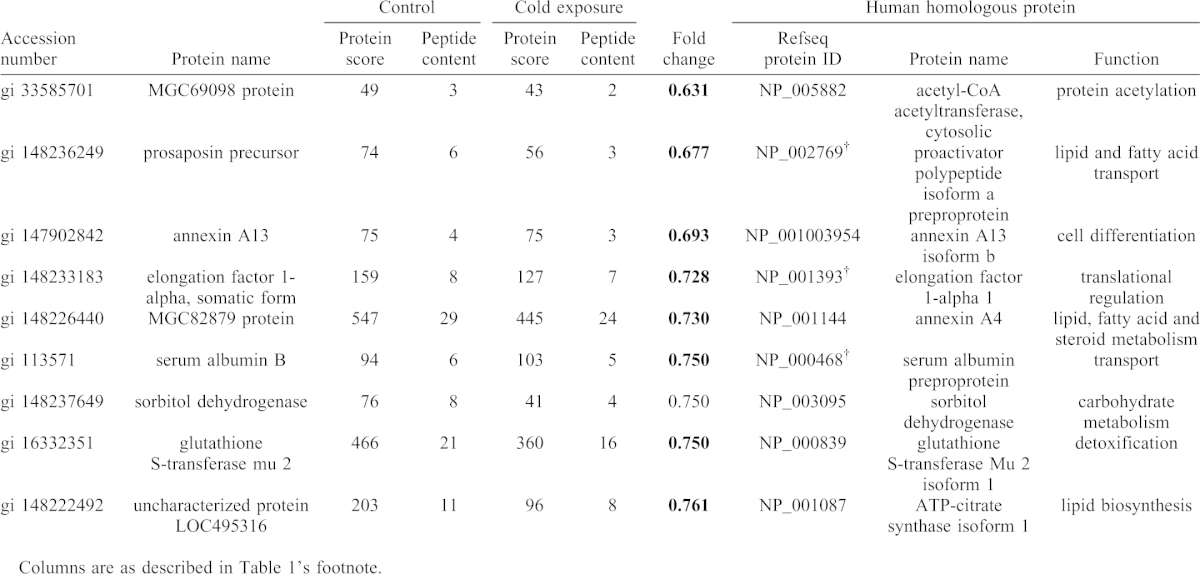
Downregulated proteins in cold-exposed *X. laevis* liver (group 3).

**Table 4.**
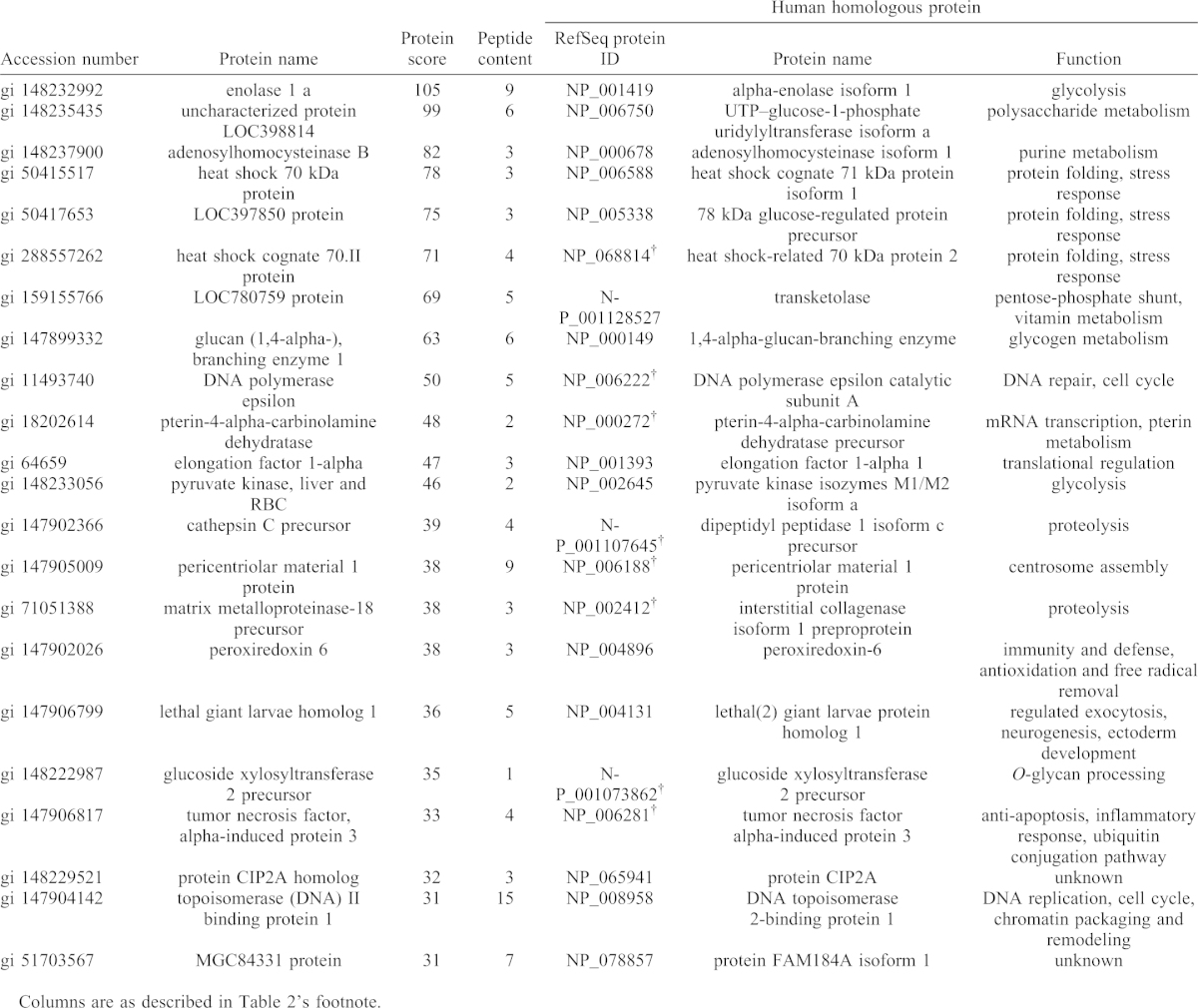
Proteins below detection limit in cold-exposed *X. laevis* liver (group 4).

### Validation of proteome data and GO and pathway analysis by metabolite measurements

In the GO analysis, the ‘tyrosine catabolic process’ and ‘l-phenylalanine catabolic process’ were significantly enriched only under cold exposure (Fig. 4B). In addition, levels of fumarylacetoacetase, which is necessary for metabolism of phenylalanine and tyrosine, tended to increase in cold exposure (Table 1). Generally, the liver metabolizes aromatic amino acids, including phenylalanine and tyrosine but not tryptophan (Dejong et al., 2007). Therefore, levels of these free amino acid levels in the liver were compared between the cold-exposure and control groups (Fig. 7A). Phenylalanine and tyrosine levels significantly decreased in cold exposure, whereas the tryptophan level did not change.

**Fig. 6.**
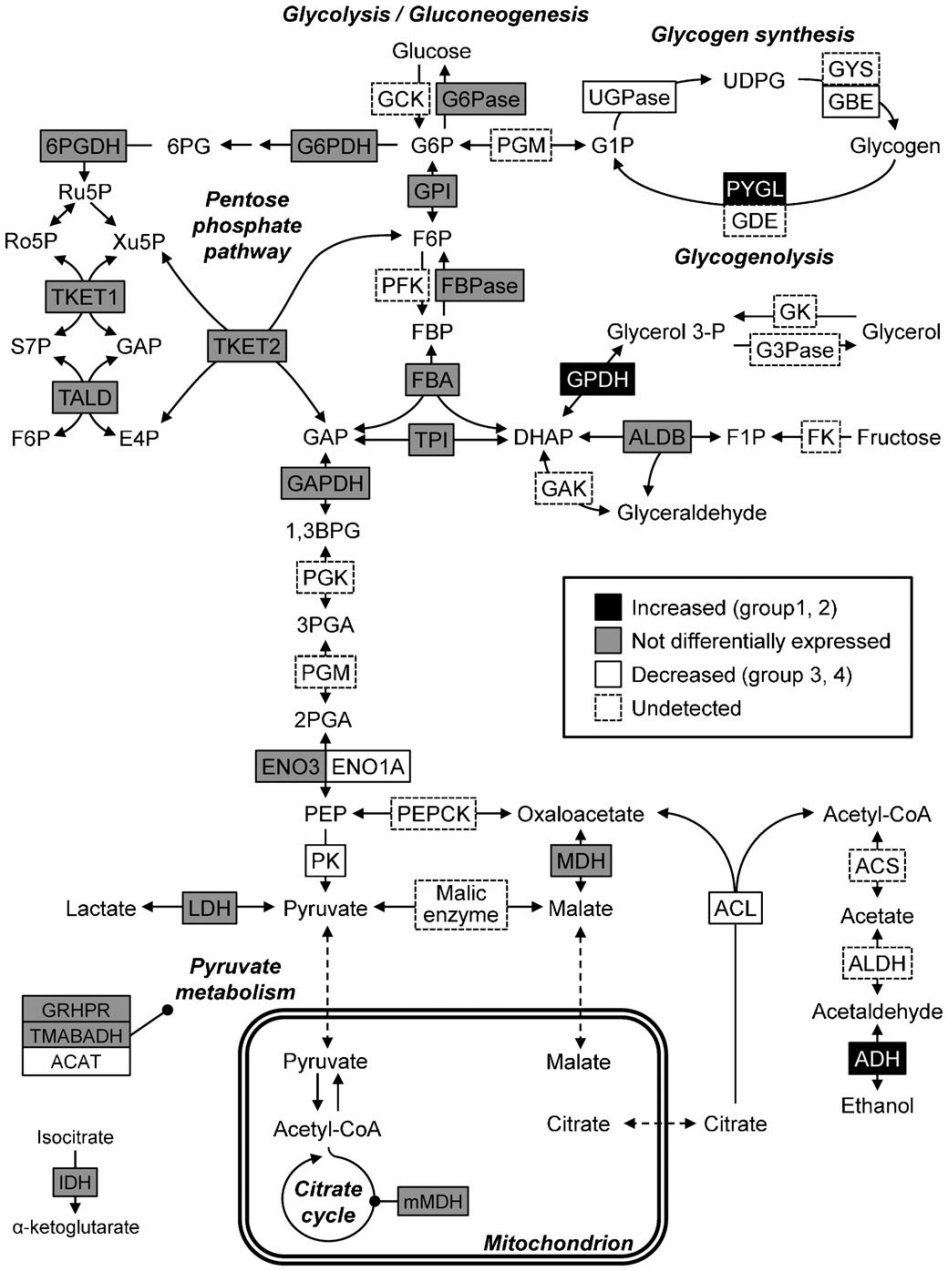
Cold-exposure-induced changes in protein abundance associated with carbohydrate metabolism. Substrates and enzymes are as follows: (glycolysis/glyconeogenesis) G6P, glucose 6-phosphate; F6P, fructose 6-phosphate; FBP, fructose 1,6-bisphosphate; GAP, glyceraldehyde 3-phosphate; DHAP, dihydroxyacetone phosphate; 1,3BPG, 1,3-bisphosphoglyceric acid; 3PGA, 3-phosphoglyceric acid; 2PGA, 2-phosphoglyceric acid; PEP, Phosphoenolpyruvate; GCK, glucokinase; G6Pase, glucose-6-phaosphatase; GPI, glucose-6-phosphate isomerase; PFK, phosphofructokinase; FBPase, fructose-1,6-bisphosphatase; FBA, fructose 1,6-bisphosphate aldorase; TPI, triosephosphate isomerase; GAPDH, glyceraldehyde-3-phosphate dehydrogenase; PGK, phosphoglyceric acid kinase; ENO, enolase; PEPCK, phosphoenolpyruvate carboxykinase; PK, pyruvate kinase; MDH, malate dehydrogenase; LDH, lactate dehydrogenase; ACL, ATP-citrate synthase (ATP-citrate lyase); ACS, acetyl-CoA synthase; ALDH, aldehyde dehydrogenase; ADH, alcohol dehydrogenase; (pentose phosphate pathway) 6PG, 6-phosphogluconate; Ro5P, ribose 5-phosphate; Ru5P, ribulose 5-phosphate; Xu5P, xylulose 5-phosphate; S7P, sedoheptulose 7-phosphate; E4P, erythrose 4-phosphate; G6PDH, glucose-6-phosphate dehydrogenase; 6PGDH, 6-phosphogluconate dehydrogenase; TKET, transketolase; TALD, transaldolase; (glycogen metabolism) G1P, glucose 1-phosphate; UDPG, uridine diphosphate glucose; PGM, phosphoglucomutase; UGPase, UDP-glucose pyrophosphorylase; GYS, glycogen synthase; PYGL, glycogen phosphorylase; GBE, glycogen branching enzyme; GDE, glycogen debranching enzyme; (others) Glycerol-3-P, glycerol 3- phosphate; F1P, fructose 1-phosphate; GK, glycerol kinase; G3Pase, glycerol-3-phosphatase; GPDH, glycerol-3-phosphate dehydrogenase; FK, fructokinase; ALDB, fructose-bisphosphate aldolase B; GAK, glyceraldehyde kinase; GRHPR, glyoxylate reductase/hydroxypyruvate reductase; TMABADH, 4-trimethylaminobutyraldehyde dehydrogenase; ACAT, acetyl-CoA acetyltransferase; mMDH, mitochondrial MDH; IDH, isocitrate dehydrogenase. Modified from portions of KEGG pathway map for ‘glycolysis/gluconeogenesis’ (00010) and ‘Pentose phosphate pathway’ (00030).

**Fig. 7.**
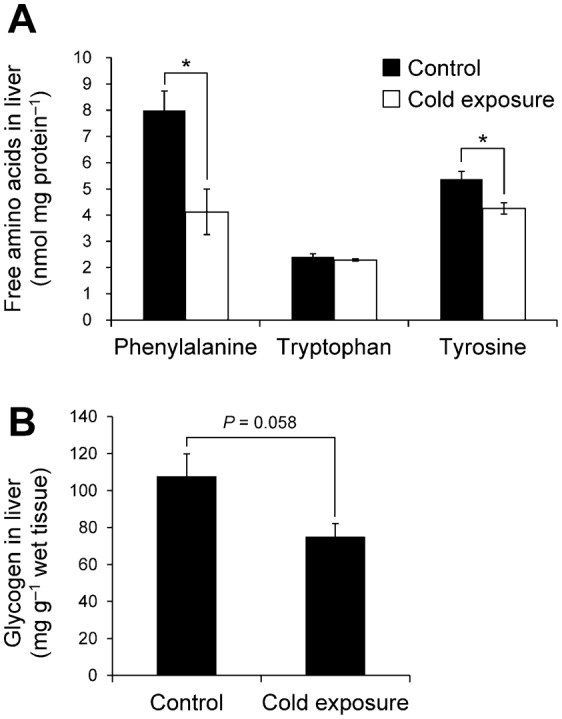
Comparisons of free aromatic amino acid levels and glycogen levels in the liver between the control and cold-exposure conditions. (A) Free amino acids in the liver (*n* = 3). (B) Glycogen in the liver (*n* = 4). Each bar represents the s.e.m. *P<0.05 by Student's *t*-test.

Fig. 6 shows cold-exposure-induced changes in protein abundance associated with carbohydrate metabolic pathways. Glycogen phosphorylase (PYGL), which catalyses the breakdown of glycogen, was increased under cold exposure (Fig. 6). Levels of 1,4-alpha-glucan branching enzyme (GBE) and UTP-glucose-1-phosphate uridylyltransferase (UDP-glucose pyrophosphorylase, UGPase), which participate in glycogen synthesis from glucose, were decreased (Fig. 6). Consequently, it is likely that synthesis of glycogen was downregulated and breakdown of glycogen was upregulated by cold exposure. Thus, we compared liver glycogen levels between the cold-exposure and control groups. The liver glycogen level tended to decrease to 70% of the control level upon cold exposure (Fig. 7B).

**Fig. 8.**
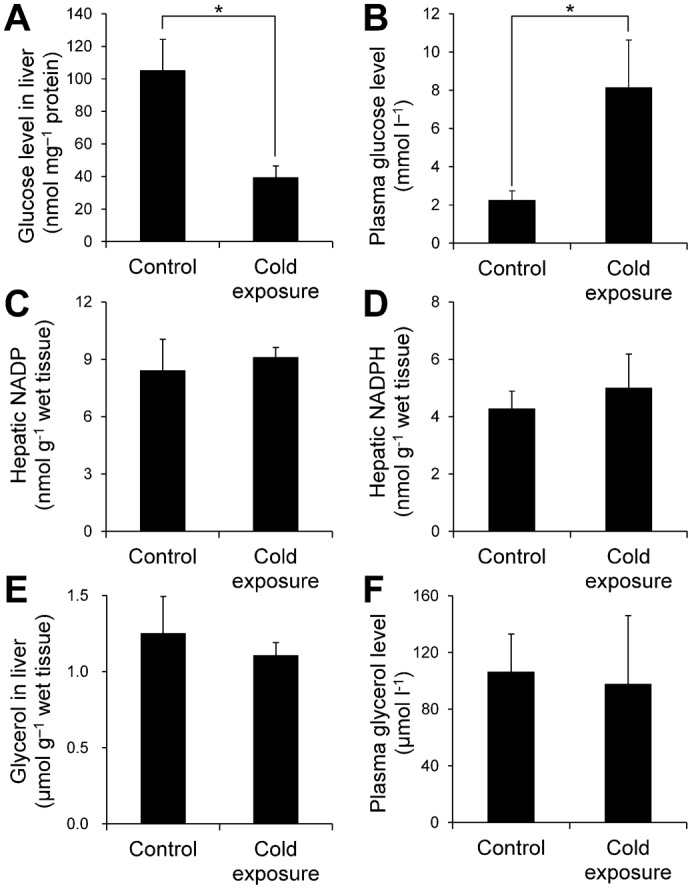
Comparisons of glucose, glycerol, NADP, and NADPH levels in the liver and/or plasma between the control and cold-exposure conditions. (A,B) Glucose levels in the liver (*n* = 3) and plasma (*n* = 5). (C,D) NADP and NADPH levels in the liver (*n* = 4). (E,F) Glycerol levels in the liver (*n* = 4) and plasma (*n* = 4). Each bar represents the s.e.m. *P<0.05 by Student's *t*-test.

Glycogen is broken down into glucose-1-phosphate (G1P) by PYGL and is subsequently converted to glucose 6-phosphate (G6P) by phosphoglucomutase (PGM). The resulting G6P is used in several metabolic pathways: (1) it is used in glucose production to supply energy for the body; (2) it fuels downstream glycolysis to produce energy in the form of ATP and NADH; (3) it is the starting substrate for the oxidative arm of the pentose phosphate pathway (PPP); (4) it may be used for biosynthesis of other metabolites. We examined whether liver glycogen was broken down for these pathways as follows:

(1) Glucose production: increased PYGL and decreased hepatic glycogen were observed (Figs 6, 7B). PYGL is upregulated in winter freeze-tolerant wood frogs (*Rana sylvatica*), which produce glucose from glycogen as a cryoprotectant in freezing (Kiss et al., 2011). When hepatic glucose production is increased, glucose levels in both liver and plasma are elevated because the glucose moves to the bloodstream across the hepatocyte membrane, mainly through facilitated diffusion through the glucose transporter (Nordlie et al., 1999). Therefore, we compared liver and plasma glucose levels between the control and cold-exposure conditions. The liver glucose level was significantly decreased to half of the level in the control under the cold-exposure condition (Fig. 8A). In contrast, the plasma glucose level significantly increased in the cold-exposure condition (approximately 3.5 fold) (Fig. 8B).

(2) Glycolysis: the level of pyruvate kinase (PK), which catalyzes the last step in the process of glycolysis by metabolising glucose to pyruvate with a net gain of two ATP and two NADH molecules per glucose molecule, was decreased (Fig. 6).

(3) PPP: the ‘pentose phosphate pathway’ was commonly associated with the liver proteome in the control condition and under cold exposure (Fig. 4C). In addition, the ‘pentose biosynthetic process’ was significantly enriched only in under exposure (Fig. 4B). These results suggest that G6P derived from glycogen may be used for PPP. The oxidative phase of PPP generates NADPH from NADP (Wamelink et al., 2008). In *X. laevis*, cold exposure causes hepatic iron accumulation as a result of heme degradation following erythrocyte destruction (Maekawa et al., 2012). Iron accumulation is associated with free radical production and elevates oxidative stress (Bacon and Britton, 1990). Cellular NADPH is important for tolerance to oxidative stress and maintenance of cellular redox homeostasis. Therefore, hepatic NADP and NADPH levels were compared between the control and cold-exposure conditions and found to be unaffected by cold exposure (Fig. 8C,D). The hepatic NADPH/NADP ratio was also not changed (data not shown).

(4) Biosynthesis of other metabolites: in rainbow smelt (*Osmerus mordax*), glycerol accumulation in the plasma is induced by low temperature (Driedzic et al., 2006). Some freeze-tolerant terrestrial anurans such as grey tree frogs (*Hyla versicolo*r and *Hyla chrysoscelis*) also produce copious quantities of glycerol as the cryoprotectant (Schmid, 1982; Irwin and Lee, 2003). These species use liver glycogen as the carbon source for glycerol synthesis. In this synthetic pathway, dihydroxyacetone phosphate (DHAP) is converted to glycerol 3-phosphate (G3P) and subsequently to glycerol via reactions catalysed by glycerol-3-phosphate dehydrogenase (GPDH) and glycerol-3-phosphatase (G3Pase), respectively. In our proteomic analysis, GPDH levels increased under cold exposure (Fig. 6), which suggests that glycogen may be used for glycerol production. To test this possibility, glycerol levels in the liver and plasma were compared between the control and cold-exposure conditions. Glycerol levels in both the liver and plasma were not changed by cold exposure (Fig. 8E,F).

## Discussion

Here, using a proteomics approach, we demonstrated the protein abundance profile of *X. laevis* liver after cold exposure. One hundred and forty-five proteins were identified from the soluble fraction of whole liver extract by trypsin digestion followed by LC–MS/MS without prior fractionation of proteins; 58 of these proteins were differentially expressed proteins (upregulated, 27; downregulated, 31). No GO:BP terms, however, were significantly enriched in these protein lists, presumably due to the small number of proteins. In recent years, global proteomics approaches have been widely used to characterize a number of tissue proteomes, including liver (Falcón-Pérez et al., 2010). Pre-fractionation of protein samples by one-dimensional electrophoresis and/or liquid chromatography has enabled the identification of more than 7000 proteins in the mouse liver (Shi et al., 2007; Lai et al., 2008). By the application of these methods, the number of identified proteins will be increased and enable GO and pathway analysis.

We have shown that the number of mature erythrocytes and accumulation of iron catalyzed from heme decrease in the liver within 24 hours after cold exposure (5°C), which reflects the enhanced destruction of erythrocytes in the liver (Maekawa et al., 2012). The enhanced hepatic destruction of erythrocytes is thought to increase the protein levels of haemoglobin subunits in the liver. Consistent with a previous finding, the protein levels of haemoglobin subunits in the liver tended to increase within 24 hours after cold exposure in our proteomic analysis (Table 1), which suggests that the results of the LC–MS/MS analysis in our study reflect physiological responses as changes in protein abundance. The peripheral erythrocyte count remains low during cold exposure (5 days) because of the hepatic confinement of newly produced erythrocytes (Maekawa et al., 2012). Some of the differentially expressed liver proteins may play a role in this phenomenon. Long-term exposure to 10°C (153 days) also causes chronic pancytopenia in *X. laevis* (Maekawa et al., 2012). Our proteomics approach is probably applicable to the investigation of the response to cold over a longer period.

Consistent with the increase in fumarylacetoacetase and cold-exposure-specific GO:BP terms (‘tyrosine catabolic process’ and ‘l-phenylalanine catabolic process’), free phenylalanine and tyrosine levels in the liver decreased (Table 1, Figs 4B, 5A). This finding suggests that amino acids were catabolized for energy, ketogenesis, and/or gluconeogenesis, and it shows the utility of a proteomics approach including GO analysis in *X. laevis*.

**Fig. 9.**
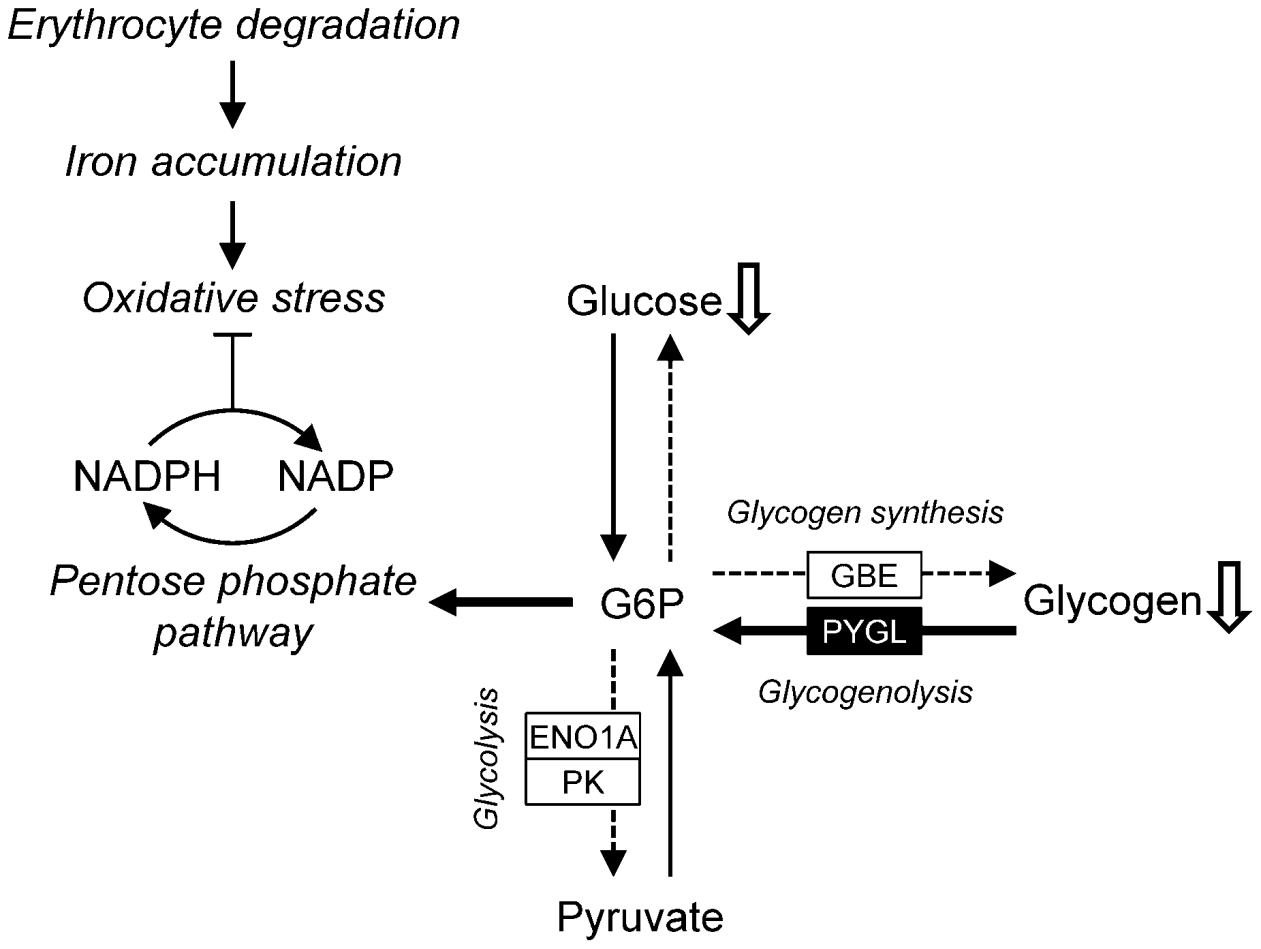
Schematic models of the early response to cold exposure in the liver of *X. laevis*. In cold exposure, hepatic glycogen and glucose are thought to be used in the pentose phosphate pathway for NADPH supply rather than in energy production through glycolysis. This mechanism suppresses oxidative stress derived from iron accumulation caused by hepatic erythrocyte degradation. Abbreviations for the substrate and enzymes are shown in Fig. 6.

Increased PYGL and decreased hepatic glycogen were observed (Table 2, Figs 6, 7B). PYGL is increased in winter freeze-tolerant wood frogs (*Rana sylvatica*) that produce glucose as a cryoprotectant from hepatic glycogen upon freezing (Kiss et al., 2011). Rainbow smelt (*Osmerus mordax*) and some freeze-tolerant terrestrial anurans such as grey tree frogs (*Hyla versicolor* and *Hyla chrysoscelis*) produce glycerol as a cryoprotectant from hepatic glycogen in winter (Driedzic et al., 2006; Schmid, 1982; Irwin and Lee, 2003). Therefore, we considered that glycogen breakdown in the liver of *X. laevis* results in production of glucose or glycerol. The glucose level in the liver, however, was decreased and liver and plasma glycerol levels were not changed (Fig. 7A,E,F). Therefore, glycogen was utilized in neither glucose nor glycerol production. Levels of PK, which participates in glycolysis, were decreased upon cold exposure, which suggests that glycolysis was inhibited by cold exposure and that G6P derived from glycogen was not used for energy production. Therefore, it is possible that the breakdown of glycogen is utilized in other pathways. Even though cold-exposed *X. laevis* presents hepatic iron accumulation that causes oxidative stress, levels of NADPH, which is consumed for protection against oxidative damage, were not decreased (Fig. 7C). This finding suggests that G6P derived from glycogen may be utilized for NADPH production by PPP for protection against oxidative damage (Fig. 9). Considering that cold-induced apoptosis in cultured rat hepatocytes and liver endothelial cells is mediated by reactive oxygen species (Rauen et al., 1999), provision of NADPH for protection from oxidative stress in the liver of cold-exposed *X. laevis* may provide a mechanism for coping with cold in freeze-intolerant ectothermic vertebrates.

The plasma glucose level was increased although the hepatic glucose level decreased (Fig. 7B). Considering that the liver is the only organ that actually releases glucose into the systemic circulation, our findings may reflect a decrease in circulating erythrocytes that consume glucose and decreased tissue glucose consumption resulting from decreased metabolism under low body temperature.

Levels of the endoplasmic reticulum (ER) molecular chaperone, 78-kDa glucose-regulated protein (GRP78, also known as BiP or Hspa5), were decreased under hypothermia in the liver of *X. laevis* (Table 4). GRP78 was found to be similarly decreased in freeze-tolerant wood frogs in the winter (*Rana sylvatica*) and in hypothermic rat livers (Oda et al., 2012; Kiss et al., 2011). In contrast, the content of GRP78 was found to be increased among the liver proteins of hibernating mammals (Epperson et al., 2004; Epperson et al., 2010). GRP is induced during ER stress derived from the disruption of calcium homeostasis and accumulation of unfolded proteins in the ER, and it protects the host cell against ER stress-induced cell death (Rao et al., 2002). Increased GRP expression is unique to hibernating animals and appears to be important.

This study presents the first system-wide screen of hepatic proteins in *X. laev*is. Low environmental temperature (i.e. low body temperature in *X. laevis*) induced modulation of the hepatic proteome, particularly in pathways associated with carbohydrate metabolism and with other functions. Regarding carbohydrate metabolism, this study suggests that utilization of hepatic glycogen for NADPH supply is associated with tolerance to cold-induced oxidative stress.

This study not only provides insight into the mechanism of tolerance to low body temperature but also demonstrates that proteomic analysis of *X. laevis* is applicable despite the lack of whole-genomic information because of the accumulation of expressed sequence tags (ESTs) in public databases. In *X. laevis*, the liver is a major site for both metabolism and haematopoiesis. Therefore, the modulation of the hepatic proteome related to metabolic pathways under low body temperature occurs at a haematopoietic site. This study provides a model for investigation of not only coping mechanisms for cold but also interactions between haematopoietic and metabolic systems.

## Supplementary Material

Supplementary Material
