# Supplementary material for: Significant modulation of the hepatic proteome induced by exposure to low temperature in Xenopus laevis
Source: Biol Open. 2013 Aug 23;2(10):1057–69. doi: 10.1242/bio.20136106 (PMC3798189; doi:10.1242/bio.20136106)
Supplement: Supplementary Material [file supp_2_10_1057__index.html]

Significant modulation of the hepatic proteome induced by exposure to low temperature in Xenopus laevis — Supplementary Material 

# Significant modulation of the hepatic proteome induced by exposure to low temperature in *Xenopus laevis*

## bio.20136106 Supplementary Material

**Files in this Data Supplement:**

- Tables S1-S4
- Table S5
