# Supplementary material for: Significant modulation of the hepatic proteome induced by exposure to low temperature in Xenopus laevis
Source: Biol Open. 2013 Aug 23;2(10):1057–69. doi: 10.1242/bio.20136106 (PMC3798189; doi:10.1242/bio.20136106)
Supplement: Supplementary Material [file supp_bio.20136106_Tables_S1-S4.doc]

**Table S1. Complete list of identified proteins**

**All proteins identified in each LC-MS/MS run are shown (289 proteins). Mascot protein scores and peptide contents of each identification and the number of detections are also included. Mixed samples from three *X*. *laevis* liver extracts of the control and cold exposure groups were analysed in triplicate (Run 1-3). Proteins identified at least twice in triplicate runs were taken to be valid (145 proteins, Table S2).**

| **Accession number** | **Protein name** | **Control** | | | | | |  | **Cold exposure** | | | | | |  | **Number of detection** | |
| --- | --- | --- | --- | --- | --- | --- | --- | --- | --- | --- | --- | --- | --- | --- | --- | --- | --- |
|  |  | **Run 1** | | **Run 2** | | **Run 3** | |  | **Run 1** | | **Run 2** | | **Run 3** | |  | **Control** | **Cold**  **exposure** |
|  |  | **Protein score** | **Peptide content** | **Protein score** | **Peptide content** | **Protein score** | **Peptide content** |  | **Protein score** | **Peptide content** | **Protein score** | **Peptide content** | **Protein score** | **Peptide content** |  |  |  |
| ***Commonly detected with valid*** | |  |  |  |  |  |  |  |  |  |  |  |  |  |  |  |  |
| gi|10197483 | aldolase B, fructose-bisphosphate | 293 | 14 | 259 | 13 | 267 | 9 |  | 275 | 12 | 258 | 9 | 244 | 9 |  | 3 | 3 |
| gi|112688 | 14-3-3-like protein | 96 | 8 | 59 | 7 | 100 | 9 |  | 96 | 5 | 100 | 6 | 110 | 8 |  | 3 | 3 |
| gi|118136396 | catalase, gene 2 | 393 | 27 | 438 | 31 | 498 | 30 |  | 404 | 25 | 490 | 26 | 455 | 27 |  | 3 | 3 |
| gi|118384 | ornithine decarboxylase 1 | 30 | 3 | 30 | 3 | 31 | 3 |  | 30 | 4 | 30 | 3 | 38 | 4 |  | 3 | 3 |
| gi|122285 | hemoglobin subunit alpha-1 | 265 | 43 | 338 | 48 | 303 | 42 |  | 403 | 65 | 371 | 63 | 400 | 61 |  | 3 | 3 |
| gi|147898618 | L-lactate dehydrogenase A chain | 38 | 2 | 91 | 3 | 66 | 3 |  | 98 | 4 | 53 | 4 | 74 | 3 |  | 3 | 3 |
| gi|147898737 | Transketolase | 110 | 5 | 76 | 4 | 81 | 7 |  | 50 | 5 | 36 | 2 | 70 | 5 |  | 3 | 3 |
| gi|147899575 | prostaglandin D2 synthase, hematopoietic a | 325 | 15 | 284 | 15 | 332 | 17 |  | 315 | 13 | 304 | 14 | 284 | 16 |  | 3 | 3 |
| gi|147900590 | argininosuccinate synthase | 446 | 30 | 450 | 28 | 379 | 24 |  | 322 | 22 | 431 | 27 | 288 | 24 |  | 3 | 3 |
| gi|147900682 | selenium-binding protein 1-B | 109 | 9 | 80 | 3 | 119 | 9 |  | 42 | 5 | 98 | 7 | 53 | 5 |  | 3 | 3 |
| gi|147902535 | MGC83388 protein | 32 | 1 | 40 | 2 | 61 | 2 |  | 56 | 2 | 36 | 2 | 34 | 1 |  | 3 | 3 |
| gi|147902599 | uncharacterized protein LOC398893 | 158 | 4 | 109 | 6 | 139 | 6 |  | 31 | 2 | 69 | 3 | 113 | 4 |  | 3 | 3 |
| gi|147902603 | hemoglobin subunit alpha-2 | 216 | 41 | 252 | 45 | 240 | 37 |  | 322 | 58 | 314 | 57 | 318 | 56 |  | 3 | 3 |
| gi|147905276 | transaldolase 1 | 110 | 4 | 153 | 9 | 92 | 8 |  | 83 | 5 | 72 | 6 | 66 | 4 |  | 3 | 3 |
| gi|147906883 | hemoglobin subunit beta-1 | 388 | 40 | 400 | 34 | 421 | 39 |  | 443 | 60 | 472 | 62 | 491 | 53 |  | 3 | 3 |
| gi|147907224 | 6-phosphogluconate dehydrogenase, decarboxylating | 57 | 2 | 124 | 5 | 65 | 5 |  | 27 | 1 | 70 | 4 | 28 | 2 |  | 3 | 3 |
| gi|147907284 | betaine--homocysteine S-methyltransferase 1 | 231 | 16 | 270 | 20 | 297 | 20 |  | 230 | 13 | 201 | 10 | 229 | 16 |  | 3 | 3 |
| gi|148223115 | fumarylacetoacetase | 98 | 3 | 84 | 5 | 101 | 7 |  | 121 | 6 | 104 | 6 | 105 | 8 |  | 3 | 3 |
| gi|148223127 | mg:bb02e05 | 182 | 9 | 195 | 11 | 138 | 7 |  | 176 | 7 | 148 | 6 | 136 | 10 |  | 3 | 3 |
| gi|148224415 | L-lactate dehydrogenase B chain | 52 | 5 | 112 | 6 | 87 | 6 |  | 115 | 6 | 71 | 7 | 89 | 4 |  | 3 | 3 |
| gi|148226440 | MGC82879 protein | 539 | 28 | 547 | 29 | 526 | 23 |  | 363 | 23 | 445 | 24 | 372 | 24 |  | 3 | 3 |
| gi|148227690 | lactate dehydrogenase A | 266 | 15 | 287 | 14 | 266 | 11 |  | 285 | 13 | 252 | 10 | 285 | 10 |  | 3 | 3 |
| gi|148228255 | aldehyde dehydrogenase 1 family, member A1 | 336 | 21 | 390 | 21 | 321 | 16 |  | 256 | 19 | 289 | 17 | 259 | 15 |  | 3 | 3 |
| gi|148229158 | transketolase-like 2 | 131 | 9 | 83 | 8 | 152 | 7 |  | 90 | 8 | 83 | 7 | 129 | 12 |  | 3 | 3 |
| gi|148229471 | glucose-6-phosphate dehydrogenase | 42 | 4 | 47 | 3 | 53 | 5 |  | 51 | 3 | 62 | 2 | 102 | 4 |  | 3 | 3 |
| gi|148229659 | uncharacterized protein LOC379555 | 176 | 10 | 200 | 8 | 196 | 7 |  | 128 | 9 | 167 | 7 | 244 | 7 |  | 3 | 3 |
| gi|148230238 | homogentisate 1,2-dioxygenase | 94 | 6 | 56 | 4 | 88 | 6 |  | 48 | 5 | 35 | 3 | 101 | 7 |  | 3 | 3 |
| gi|148230659 | glutamate dehydrogenase 1 | 76 | 4 | 76 | 3 | 124 | 7 |  | 72 | 3 | 38 | 5 | 53 | 4 |  | 3 | 3 |
| gi|148231271 | aldehyde dehydrogenase 9 family, member A1 | 553 | 25 | 479 | 24 | 501 | 29 |  | 417 | 24 | 407 | 17 | 468 | 23 |  | 3 | 3 |
| gi|148232240 | isocitrate dehydrogenase 1 | 32 | 6 | 41 | 6 | 73 | 5 |  | 50 | 6 | 64 | 7 | 66 | 6 |  | 3 | 3 |
| gi|148232264 | argininosuccinate lyase | 267 | 12 | 201 | 13 | 199 | 10 |  | 93 | 5 | 154 | 8 | 131 | 8 |  | 3 | 3 |
| gi|148233713 | arginase 1 | 414 | 20 | 328 | 17 | 399 | 14 |  | 292 | 18 | 358 | 14 | 374 | 16 |  | 3 | 3 |
| gi|148234425 | glucose-6-phosphate isomerase | 39 | 4 | 33 | 5 | 91 | 7 |  | 46 | 3 | 34 | 3 | 49 | 5 |  | 3 | 3 |
| gi|148234619 | carbamoyl-phosphate synthase 1, mitochondrial | 359 | 24 | 313 | 21 | 366 | 27 |  | 247 | 15 | 410 | 21 | 313 | 18 |  | 3 | 3 |
| gi|148234947 | UDP-glucose pyrophosphorylase 2 | 152 | 7 | 135 | 10 | 202 | 11 |  | 161 | 9 | 216 | 11 | 153 | 11 |  | 3 | 3 |
| gi|148235865 | cold-inducible RNA-binding protein B | 29 | 1 | 43 | 2 | 50 | 4 |  | 58 | 3 | 40 | 2 | 50 | 3 |  | 3 | 3 |
| gi|148236091 | fructose-1,6-bisphosphatase 1 | 60 | 4 | 57 | 4 | 62 | 4 |  | 102 | 4 | 92 | 4 | 68 | 4 |  | 3 | 3 |
| gi|148236249 | prosaposin (variant Gaucher disease and variant metachromatic leukodystrophy) precursor | 74 | 6 | 61 | 2 | 44 | 2 |  | 55 | 2 | 56 | 2 | 36 | 3 |  | 3 | 3 |
| gi|148236351 | triosephosphate isomerase | 56 | 4 | 109 | 5 | 77 | 3 |  | 115 | 3 | 118 | 3 | 119 | 4 |  | 3 | 3 |
| gi|148237546 | MGC83376 protein | 261 | 19 | 313 | 20 | 291 | 18 |  | 147 | 8 | 189 | 14 | 186 | 8 |  | 3 | 3 |
| gi|16332351 | glutathione S-transferase | 466 | 18 | 408 | 21 | 414 | 20 |  | 352 | 16 | 360 | 14 | 330 | 15 |  | 3 | 3 |
| gi|21952442 | glutathione S-transferase mu | 147 | 6 | 122 | 6 | 136 | 8 |  | 146 | 6 | 130 | 7 | 139 | 6 |  | 3 | 3 |
| gi|291290905 | hemoglobin, gamma G | 211 | 13 | 198 | 16 | 201 | 16 |  | 232 | 20 | 244 | 24 | 263 | 22 |  | 3 | 3 |
| gi|32450751 | LOC398623 protein | 290 | 14 | 267 | 13 | 272 | 8 |  | 272 | 13 | 197 | 10 | 210 | 9 |  | 3 | 3 |
| gi|4586546 | aldehyde dehydrogenase 1A | 377 | 23 | 367 | 23 | 351 | 17 |  | 258 | 17 | 250 | 15 | 292 | 18 |  | 3 | 3 |
| gi|55824753 | LOC495840 protein | 108 | 10 | 143 | 13 | 175 | 13 |  | 91 | 10 | 138 | 11 | 105 | 9 |  | 3 | 3 |
| gi|64647 | Cu-Zn superoxide dismutase C-terminal fragment (150AA) | 116 | 4 | 128 | 3 | 106 | 4 |  | 92 | 5 | 94 | 3 | 89 | 6 |  | 3 | 3 |
| gi|77748240 | MGC82659 protein | 162 | 5 | 173 | 6 | 166 | 5 |  | 181 | 6 | 135 | 5 | 184 | 6 |  | 3 | 3 |
| gi|9910617 | allantoicase | 82 | 6 | 41 | 5 | 89 | 6 |  | 110 | 8 | 53 | 5 | 84 | 7 |  | 3 | 3 |
| gi|1065161 | superoxide dismutase [Cu-Zn] B | 159 | 5 | 171 | 4 | 164 | 5 |  | 139 | 5 | - | - | 123 | 4 |  | 3 | 2 |
| gi|113571 | serum albumin B | 74 | 3 | 67 | 3 | 94 | 6 |  | - | - | 75 | 3 | 103 | 5 |  | 3 | 2 |
| gi|147902842 | annexin A13 | 56 | 4 | 72 | 3 | 75 | 3 |  | - | - | 48 | 3 | 75 | 3 |  | 3 | 2 |
| gi|147906522 | potassium voltage-gated channel, Shab-related subfamily, member 2 | 39 | 7 | 39 | 8 | 36 | 7 |  | - | - | 38 | 12 | 31 | 10 |  | 3 | 2 |
| gi|148222492 | uncharacterized protein LOC495316 | 203 | 11 | 155 | 11 | 185 | 10 |  | 82 | 8 | - | - | 96 | 7 |  | 3 | 2 |
| gi|308153262 | family with sequence similarity 64, member A | 36 | 2 | 38 | 3 | 32 | 2 |  | - | - | 41 | 3 | 33 | 2 |  | 3 | 2 |
| gi|54037970 | LOC495053 protein | 41 | 8 | 35 | 5 | 40 | 6 |  | - | - | 27 | 6 | 52 | 13 |  | 3 | 2 |
| gi|147898869 | purine nucleoside phosphorylase | 65 | 6 | 90 | 7 | 69 | 7 |  | 64 | 3 | 84 | 5 | - | - |  | 3 | 2 |
| gi|148230001 | GTP cyclohydrolase 1 feedback regulatory protein | 53 | 2 | 51 | 2 | 53 | 1 |  | 54 | 3 | 54 | 3 | - | - |  | 3 | 2 |
| gi|148232311 | nucleolar and spindle-associated protein 1-A | 28 | 3 | 34 | 3 | 28 | 2 |  | 27 | 1 | 29 | 1 | - | - |  | 3 | 2 |
| gi|148237649 | sorbitol dehydrogenase | 76 | 8 | 73 | 6 | 58 | 5 |  | 41 | 4 | 39 | 4 | - | - |  | 3 | 2 |
| gi|1703127 | actin, cytoplasmic type 8 | 227 | 12 | 237 | 16 | 202 | 12 |  | 182 | 12 | 130 | 9 | - | - |  | 3 | 2 |
| gi|32450739 | alanine-glyoxylate aminotransferase | 113 | 6 | 28 | 3 | 72 | 8 |  | 39 | 3 | 43 | 3 | - | - |  | 3 | 2 |
| gi|6225751 | nucleoside diphosphate kinase A1 | 66 | 4 | 58 | 3 | 53 | 2 |  | 81 | 4 | 82 | 4 | - | - |  | 3 | 2 |
| gi|11035016 | SWI/SNF related, matrix associated, actin dependent regulator of chromatin, subfamily a, member 5 | - | - | 52 | 7 | 54 | 8 |  | 34 | 6 | 36 | 6 | 38 | 8 |  | 2 | 3 |
| gi|11385422 | serine/threonine-protein kinase atr | - | - | 32 | 3 | 57 | 7 |  | 57 | 5 | 40 | 5 | 47 | 5 |  | 2 | 3 |
| gi|120577551 | glyoxylate reductase/hydroxypyruvate reductase-like protein | - | - | 163 | 11 | 118 | 7 |  | 99 | 8 | 98 | 5 | 146 | 7 |  | 2 | 3 |
| gi|147899037 | malate dehydrogenase 2, NAD (mitochondrial) | 45 | 4 | - | - | 55 | 5 |  | 96 | 5 | 59 | 4 | 73 | 5 |  | 2 | 3 |
| gi|148222055 | similar to carbonic anhydrase II | 54 | 2 | - | - | 37 | 1 |  | 57 | 1 | 54 | 1 | 29 | 1 |  | 2 | 3 |
| gi|148223641 | catalase, gene 2 | - | - | 425 | 31 | 495 | 28 |  | 381 | 24 | 480 | 25 | 413 | 25 |  | 2 | 3 |
| gi|148223868 | malate dehydrogenase, cytoplasmic | 48 | 2 | 27 | 2 | - | - |  | 32 | 1 | 31 | 1 | 44 | 2 |  | 2 | 3 |
| gi|148226821 | cofilin-1-B | 66 | 2 | 39 | 1 | - | - |  | 49 | 1 | 55 | 2 | 77 | 3 |  | 2 | 3 |
| gi|148232692 | MGC80314 protein | - | - | 48 | 2 | 33 | 2 |  | 43 | 1 | 27 | 2 | 61 | 1 |  | 2 | 3 |
| gi|148233183 | elongation factor 1-alpha, somatic form | 156 | 8 | 159 | 6 | - | - |  | 127 | 4 | 109 | 7 | 100 | 4 |  | 2 | 3 |
| gi|148236059 | aldo-keto reductase family 1, member C1 (dihydrodiol dehydrogenase 1; 20-alpha (3-alpha)-hydroxysteroid dehydrogenase) | 44 | 4 | - | - | 39 | 3 |  | 35 | 2 | 56 | 3 | 34 | 3 |  | 2 | 3 |
| gi|7416136 | regucalcin (senescence marker protein-30) | 109 | 8 | - | - | 105 | 5 |  | 75 | 6 | 59 | 6 | 69 | 4 |  | 2 | 3 |
| gi|147904364 | epoxide hydrolase 2, cytoplasmic | 39 | 8 | - | - | 45 | 4 |  | - | - | 42 | 5 | 51 | 6 |  | 2 | 2 |
| gi|147904511 | enolase 3 | - | - | 66 | 8 | 51 | 6 |  | - | - | 72 | 7 | 43 | 6 |  | 2 | 2 |
| gi|147906867 | eukaryotic translation elongation factor 2, gene 1 | 51 | 6 | - | - | 29 | 4 |  | 51 | 3 | - | - | 33 | 3 |  | 2 | 2 |
| gi|33416812 | lymphocyte cytosolic protein 1 (L-plastin) | - | - | 48 | 3 | 38 | 3 |  | 42 | 4 | - | - | 64 | 2 |  | 2 | 2 |
| gi|33585701 | MGC69098 protein | 34 | 2 | - | - | 49 | 3 |  | 31 | 1 | - | - | 43 | 2 |  | 2 | 2 |
| gi|62740095 | LOC733209 protein | 35 | 8 | 31 | 5 | - | - |  | - | - | 29 | 5 | 33 | 7 |  | 2 | 2 |
| ***Detected in control with valid*** | |  |  |  |  |  |  |  |  |  |  |  |  |  |  |  |  |
| ***--- Control-specific*** | |  |  |  |  |  |  |  |  |  |  |  |  |  |  |  |  |
| gi|147902026 | peroxiredoxin 6 | 29 | 3 | 38 | 3 | 37 | 4 |  | - | - | - | - | - | - |  | 3 | 0 |
| gi|148232992 | enolase 1 a | 105 | 9 | 69 | 8 | 51 | 6 |  | - | - | - | - | - | - |  | 3 | 0 |
| gi|50417653 | LOC397850 protein | 75 | 3 | 51 | 2 | 45 | 2 |  | - | - | - | - | - | - |  | 3 | 0 |
| gi|64659 | elongation factor 1-alpha (454 AA) | 36 | 3 | 29 | 2 | 47 | 3 |  | - | - | - | - | - | - |  | 3 | 0 |
| gi|11493740 | DNA polymerase epsilon | 46 | 9 | - | - | 50 | 5 |  | - | - | - | - | - | - |  | 2 | 0 |
| gi|147899332 | glucan (1,4-alpha-), branching enzyme 1 (glycogen branching enzyme, Andersen disease, glycogen storage disease type IV) | 40 | 8 | 63 | 6 | - | - |  | - | - | - | - | - | - |  | 2 | 0 |
| gi|147902366 | cathepsin C precursor | - | - | 39 | 4 | 39 | 2 |  | - | - | - | - | - | - |  | 2 | 0 |
| gi|147904142 | topoisomerase (DNA) II binding protein 1 | 31 | 15 | - | - | 29 | 13 |  | - | - | - | - | - | - |  | 2 | 0 |
| gi|147905009 | pericentriolar material 1 protein | - | - | 29 | 7 | 38 | 9 |  | - | - | - | - | - | - |  | 2 | 0 |
| gi|147906799 | lethal giant larvae homolog 1 | 36 | 5 | - | - | 28 | 6 |  | - | - | - | - | - | - |  | 2 | 0 |
| gi|147906817 | tumor necrosis factor, alpha-induced protein 3 | - | - | 27 | 3 | 33 | 4 |  | - | - | - | - | - | - |  | 2 | 0 |
| gi|148222987 | glucoside xylosyltransferase 2 precursor | - | - | 32 | 1 | 35 | 1 |  | - | - | - | - | - | - |  | 2 | 0 |
| gi|148229521 | protein CIP2A homolog | - | - | 32 | 3 | 31 | 5 |  | - | - | - | - | - | - |  | 2 | 0 |
| gi|148233056 | pyruvate kinase, liver and RBC | 40 | 1 | 46 | 2 | - | - |  | - | - | - | - | - | - |  | 2 | 0 |
| gi|148235435 | uncharacterized protein LOC398814 | 82 | 5 | - | - | 99 | 6 |  | - | - | - | - | - | - |  | 2 | 0 |
| gi|148237900 | adenosylhomocysteinase B | 78 | 5 | 82 | 3 | - | - |  | - | - | - | - | - | - |  | 2 | 0 |
| gi|159155766 | LOC780759 protein | 69 | 5 | - | - | 68 | 6 |  | - | - | - | - | - | - |  | 2 | 0 |
| gi|18202614 | dimerization cofactor of hepatocyte nuclear factor 1-alpha | - | - | 30 | 2 | 48 | 2 |  | - | - | - | - | - | - |  | 2 | 0 |
| gi|288557262 | heat shock cognate 70.II protein | - | - | 71 | 4 | 47 | 5 |  | - | - | - | - | - | - |  | 2 | 0 |
| gi|50415517 | heat shock 70 kDa protein | 78 | 3 | 48 | 3 | - | - |  | - | - | - | - | - | - |  | 2 | 0 |
| gi|51703567 | MGC84331 protein | 31 | 7 | - | - | 29 | 5 |  | - | - | - | - | - | - |  | 2 | 0 |
| gi|71051388 | matrix metalloproteinase-18 precursor | 33 | 3 | - | - | 38 | 3 |  | - | - | - | - | - | - |  | 2 | 0 |
| gi|147906560 | male-specific lethal 3 homolog | 30 | 2 | 32 | 3 | 29 | 3 |  | - | - | - | - | 32 | 2 |  | 3 | 1 |
| ***--- Not specific*** | |  |  |  |  |  |  |  |  |  |  |  |  |  |  |  |  |
| gi|148225538 | tyrosine 3-monooxygenase/tryptophan 5-monooxygenase activation protein, epsilon polypeptide | 87 | 7 | 30 | 5 | 98 | 8 |  | - | - | - | - | 53 | 3 |  | 3 | 1 |
| gi|171846437 | LOC100158433 protein | 29 | 7 | 31 | 7 | 28 | 3 |  | - | - | - | - | 28 | 4 |  | 3 | 1 |
| gi|27882192 | glyceraldehyde-3-phosphate dehydrogenase | 139 | 5 | 81 | 7 | 68 | 4 |  | - | - | - | - | 63 | 5 |  | 3 | 1 |
| gi|147903225 | glutamic-oxaloacetic transaminase 1, soluble (aspartate aminotransferase 1) | 57 | 2 | 57 | 3 | 65 | 2 |  | 34 | 1 | - | - | - | - |  | 3 | 1 |
| gi|147903369 | ribosomal protein L6 | 29 | 1 | 40 | 2 | 29 | 1 |  | - | - | 30 | 1 | - | - |  | 3 | 1 |
| gi|148222521 | ATP citrate lyase | 166 | 10 | 126 | 10 | 126 | 8 |  | 75 | 8 | - | - | - | - |  | 3 | 1 |
| gi|148227210 | zinc finger protein 367 | 43 | 2 | 43 | 3 | 28 | 2 |  | 43 | 2 | - | - | - | - |  | 3 | 1 |
| gi|148234370 | peroxiredoxin 5 precursor | 34 | 2 | 31 | 4 | 54 | 4 |  | - | - | 43 | 2 | - | - |  | 3 | 1 |
| gi|148235689 | alpha-enolase | 157 | 11 | 85 | 11 | 75 | 8 |  | - | - | 75 | 6 | - | - |  | 3 | 1 |
| gi|27695014 | MGC64293 protein | 53 | 2 | - | - | 36 | 2 |  | - | - | - | - | 45 | 1 |  | 2 | 1 |
| gi|147902134 | aldehyde dehydrogenase 1 family, member A2 | - | - | 53 | 3 | 47 | 2 |  | - | - | 30 | 2 | - | - |  | 2 | 1 |
| gi|147904296 | uncharacterized protein LOC494651 | - | - | 31 | 3 | 30 | 3 |  | 30 | 2 | - | - | - | - |  | 2 | 1 |
| gi|148223708 | zinc finger protein 703-B | 30 | 1 | - | - | 30 | 1 |  | - | - | 32 | 2 | - | - |  | 2 | 1 |
| gi|148224943 | selenium-binding protein 1-A | 66 | 6 | - | - | 53 | 6 |  | - | - | 48 | 4 | - | - |  | 2 | 1 |
| gi|148232365 | structural maintenance of chromosomes 1A | - | - | 37 | 4 | 28 | 5 |  | - | - | 28 | 2 | - | - |  | 2 | 1 |
| gi|148234205 | aldo-keto reductase family 1, member C-like 1 | 27 | 3 | - | - | 36 | 5 |  | - | - | 46 | 3 | - | - |  | 2 | 1 |
| gi|156119465 | keratin, type II cytoskeletal | 50 | 3 | 69 | 5 | - | - |  | 51 | 3 | - | - | - | - |  | 2 | 1 |
| gi|157426935 | uncharacterized protein LOC100125664 | - | - | 50 | 4 | 64 | 5 |  | 53 | 5 | - | - | - | - |  | 2 | 1 |
| gi|28436918 | prolyl 4-hydroxylase, beta polypeptide precursor | - | - | 28 | 2 | 92 | 4 |  | 82 | 6 | - | - | - | - |  | 2 | 1 |
| gi|33411670 | E3 ubiquitin-protein ligase RNF12-A | 30 | 1 | - | - | 30 | 1 |  | - | - | 31 | 1 | - | - |  | 2 | 1 |
| gi|46249667 | LOC414586 protein | - | - | 34 | 2 | 30 | 2 |  | - | - | 43 | 2 | - | - |  | 2 | 1 |
| gi|7439974 | cold-inducible RNA-binding protein A | 39 | 2 | 31 | 3 | - | - |  | 39 | 2 | - | - | - | - |  | 2 | 1 |
| ***Detected in cold exposure with valid*** | |  |  |  |  |  |  |  |  |  |  |  |  |  |  |  |  |
| ***--- Cold exposure-specific*** | |  |  |  |  |  |  |  |  |  |  |  |  |  |  |  |  |
| gi|125858908 | Unknown (protein for IMAGE:8550378) | - | - | - | - | - | - |  | - | - | 30 | 5 | 29 | 4 |  | 0 | 2 |
| gi|147898691 | DNA-dependent protein kinase catalytic subunit | - | - | - | - | - | - |  | 29 | 8 | - | - | 33 | 6 |  | 0 | 2 |
| gi|147901600 | liver glycogen phosphorylase | - | - | - | - | - | - |  | - | - | 31 | 2 | 35 | 4 |  | 0 | 2 |
| gi|147902854 | zinc finger protein 507 | - | - | - | - | - | - |  | - | - | 31 | 5 | 27 | 4 |  | 0 | 2 |
| gi|147905626 | alcohol dehydrogenase 1C (class I), gamma polypeptide | - | - | - | - | - | - |  | - | - | 29 | 2 | 35 | 2 |  | 0 | 2 |
| gi|147905834 | lysosomal thioesterase PPT2-B precursor | - | - | - | - | - | - |  | 50 | 11 | - | - | 58 | 11 |  | 0 | 2 |
| gi|148225833 | glycerol-3-phosphate dehydrogenase 1 (soluble) | - | - | - | - | - | - |  | 54 | 3 | - | - | 38 | 2 |  | 0 | 2 |
| gi|160420189 | dopa decarboxylase (aromatic L-amino acid decarboxylase) | - | - | - | - | - | - |  | - | - | 32 | 6 | 28 | 5 |  | 0 | 2 |
| gi|3745759 | histone H4 | - | - | - | - | - | - |  | - | - | 43 | 3 | 75 | 3 |  | 0 | 2 |
| gi|37791449 | protein tyrosine phosphatase PTP-PEST | - | - | - | - | - | - |  | - | - | 28 | 4 | 37 | 2 |  | 0 | 2 |
| gi|49115587 | LOC443650 protein | - | - | - | - | - | - |  | - | - | 29 | 3 | 32 | 2 |  | 0 | 2 |
| gi|68534041 | LOC733291 protein | - | - | - | - | - | - |  | - | - | 33 | 2 | 55 | 4 |  | 0 | 2 |
| gi|147907415 | ropporin-1-like protein | - | - | - | - | - | - |  | 28 | 3 | 27 | 3 | - | - |  | 0 | 2 |
| ***--- Not specific*** | |  |  |  |  |  |  |  |  |  |  |  |  |  |  |  |  |
| gi|148230513 | adenosylhomocysteinase A | - | - | - | - | 143 | 5 |  | 79 | 3 | 106 | 4 | 95 | 4 |  | 1 | 3 |
| gi|138532 | vimentin-4 | - | - | 29 | 2 | - | - |  | 37 | 3 | - | - | 52 | 4 |  | 1 | 2 |
| gi|148222316 | uncharacterized protein LOC379202 | - | - | - | - | 55 | 3 |  | - | - | 34 | 4 | 42 | 3 |  | 1 | 2 |
| gi|148237590 | malate dehydrogenase 2, NAD (mitochondrial) | - | - | 52 | 5 | - | - |  | - | - | 80 | 6 | 104 | 7 |  | 1 | 2 |
| gi|32450581 | peptidylprolyl isomerase A (cyclophilin A) | 32 | 1 | - | - | - | - |  | 28 | 1 | 31 | 1 | - | - |  | 1 | 2 |
| gi|64580 | beta-2 globin | 184 | 11 | - | - | - | - |  | 214 | 16 | 226 | 21 | - | - |  | 1 | 2 |
| ***Detected without valid*** | |  |  |  |  |  |  |  |  |  |  |  |  |  |  |  |  |
| gi|11878220 | transcriptional repressor | - | - | 27 | 4 | - | - |  | - | - | - | - | 32 | 5 |  | 1 | 1 |
| gi|1360640 | 14-3-3 zeta protein | - | - | 66 | 6 | - | - |  | - | - | - | - | 68 | 7 |  | 1 | 1 |
| gi|147906717 | uncharacterized protein LOC379566 | - | - | - | - | 40 | 2 |  | - | - | - | - | 29 | 2 |  | 1 | 1 |
| gi|148227256 | isocitrate dehydrogenase 2 (NADP+), mitochondrial | 30 | 5 | - | - | - | - |  | - | - | - | - | 32 | 3 |  | 1 | 1 |
| gi|147903723 | nuclear/mitotic apparatus protein | 30 | 9 | - | - | - | - |  | - | - | 34 | 11 | - | - |  | 1 | 1 |
| gi|147904788 | tubulin, alpha 1a | 42 | 4 | - | - | - | - |  | 44 | 4 | - | - | - | - |  | 1 | 1 |
| gi|147905081 | glutamine-fructose-6-phosphate transaminase 2 | 31 | 2 | - | - | - | - |  | 39 | 3 | - | - | - | - |  | 1 | 1 |
| gi|147906208 | low density lipoprotein receptor-related protein 6 precursor | - | - | - | - | 32 | 6 |  | - | - | 32 | 9 | - | - |  | 1 | 1 |
| gi|148229789 | aldehyde dehydrogenase 2 family (mitochondrial) | - | - | - | - | 51 | 3 |  | - | - | 54 | 2 | - | - |  | 1 | 1 |
| gi|148230258 | glucan (1,4-alpha-), branching enzyme 1 | - | - | 55 | 6 | - | - |  | 28 | 3 | - | - | - | - |  | 1 | 1 |
| gi|148232010 | TNFAIP3 interacting protein 1 | - | - | - | - | 35 | 5 |  | - | - | 38 | 4 | - | - |  | 1 | 1 |
| gi|148232710 | MAP/microtubule affinity-regulating kinase 1 | - | - | 30 | 4 | - | - |  | 28 | 8 | - | - | - | - |  | 1 | 1 |
| gi|209156424 | Chain B, The Effect Of H3 K79 Dimethylation And H4 K20 Trimethylation On Nucleosome And Chromatin Structure | - | - | - | - | 30 | 3 |  | 69 | 4 | - | - | - | - |  | 1 | 1 |
| gi|49118496 | MGC80994 protein | - | - | - | - | 29 | 7 |  | - | - | 28 | 4 | - | - |  | 1 | 1 |
| gi|51895487 | merlin | - | - | - | - | 44 | 5 |  | 34 | 2 | - | - | - | - |  | 1 | 1 |
| gi|1017726 | BTEB | - | - | 34 | 2 | - | - |  | - | - | - | - | - | - |  | 1 | 0 |
| gi|116487529 | LOC779090 protein | 28 | 3 | - | - | - | - |  | - | - | - | - | - | - |  | 1 | 0 |
| gi|1170158 | histone H2A type 2 | - | - | 27 | 1 | - | - |  | - | - | - | - | - | - |  | 1 | 0 |
| gi|120960 | GATA-binding factor 2 | - | - | - | - | 30 | 3 |  | - | - | - | - | - | - |  | 1 | 0 |
| gi|125112 | keratin, type II cytoskeletal 8 | 63 | 4 | - | - | - | - |  | - | - | - | - | - | - |  | 1 | 0 |
| gi|1255995 | heat shock cognate 70.I | - | - | 69 | 4 | - | - |  | - | - | - | - | - | - |  | 1 | 0 |
| gi|13624776 | fructose-1,6-bisphosphatase | 74 | 3 | - | - | - | - |  | - | - | - | - | - | - |  | 1 | 0 |
| gi|138531 | RecName: Full=Vimentin-1/2 | - | - | - | - | 34 | 1 |  | - | - | - | - | - | - |  | 1 | 0 |
| gi|147898638 | histone H4 transcription factor | - | - | - | - | 29 | 1 |  | - | - | - | - | - | - |  | 1 | 0 |
| gi|147898789 | centrosomal protein 290kDa | 34 | 5 | - | - | - | - |  | - | - | - | - | - | - |  | 1 | 0 |
| gi|147900083 | tetratricopeptide repeat domain 4 | - | - | 30 | 3 | - | - |  | - | - | - | - | - | - |  | 1 | 0 |
| gi|147900139 | ERBB receptor feedback inhibitor 1 | - | - | 30 | 2 | - | - |  | - | - | - | - | - | - |  | 1 | 0 |
| gi|147900768 | MGC81887 protein | 47 | 2 | - | - | - | - |  | - | - | - | - | - | - |  | 1 | 0 |
| gi|147900881 | structural maintenance of chromosomes 3 | 30 | 3 | - | - | - | - |  | - | - | - | - | - | - |  | 1 | 0 |
| gi|147901745 | 14-3-3 protein gamma-B | 54 | 6 | - | - | - | - |  | - | - | - | - | - | - |  | 1 | 0 |
| gi|147902645 | ribosomal protein SA | - | - | - | - | 33 | 3 |  | - | - | - | - | - | - |  | 1 | 0 |
| gi|147903211 | transgelin 2 | - | - | 43 | 2 | - | - |  | - | - | - | - | - | - |  | 1 | 0 |
| gi|147903298 | aminoacylase 1, gene 1 | - | - | - | - | 27 | 3 |  | - | - | - | - | - | - |  | 1 | 0 |
| gi|147903473 | PTPRF interacting protein, binding protein 1 (liprin beta 1) | - | - | 30 | 4 | - | - |  | - | - | - | - | - | - |  | 1 | 0 |
| gi|147904268 | uncharacterized protein LOC100037235 | - | - | - | - | 31 | 2 |  | - | - | - | - | - | - |  | 1 | 0 |
| gi|147904649 | ribosomal protein, large, P0 | 44 | 4 | - | - | - | - |  | - | - | - | - | - | - |  | 1 | 0 |
| gi|147905135 | regucalcin | - | - | 120 | 8 | - | - |  | - | - | - | - | - | - |  | 1 | 0 |
| gi|147905228 | tubulin, alpha 3c | 40 | 4 | - | - | - | - |  | - | - | - | - | - | - |  | 1 | 0 |
| gi|147905376 | splicing factor 3B subunit 1 | 37 | 5 | - | - | - | - |  | - | - | - | - | - | - |  | 1 | 0 |
| gi|147905582 | eosinophil peroxidase precursor | - | - | - | - | 28 | 2 |  | - | - | - | - | - | - |  | 1 | 0 |
| gi|147905648 | uncharacterized protein LOC100036902 | - | - | 96 | 9 | - | - |  | - | - | - | - | - | - |  | 1 | 0 |
| gi|147905746 | tubulin beta-4 chain | - | - | - | - | 43 | 2 |  | - | - | - | - | - | - |  | 1 | 0 |
| gi|147906069 | cathepsin Z precursor | - | - | 43 | 1 | - | - |  | - | - | - | - | - | - |  | 1 | 0 |
| gi|147906789 | ribosomal protein S9 | - | - | - | - | 27 | 3 |  | - | - | - | - | - | - |  | 1 | 0 |
| gi|147906917 | importin 5 | - | - | 29 | 5 | - | - |  | - | - | - | - | - | - |  | 1 | 0 |
| gi|148222067 | uncharacterized protein LOC734611 | - | - | - | - | 27 | 2 |  | - | - | - | - | - | - |  | 1 | 0 |
| gi|148223453 | cytochrome P450, family 26, subfamily A, polypeptide 1 | 33 | 4 | - | - | - | - |  | - | - | - | - | - | - |  | 1 | 0 |
| gi|148223499 | tubulin beta-2 chain | - | - | - | - | 31 | 2 |  | - | - | - | - | - | - |  | 1 | 0 |
| gi|148224359 | ribosomal protein L27a | - | - | - | - | 29 | 1 |  | - | - | - | - | - | - |  | 1 | 0 |
| gi|148224766 | probable arginyl-tRNA synthetase, mitochondrial precursor | - | - | - | - | 33 | 7 |  | - | - | - | - | - | - |  | 1 | 0 |
| gi|148225278 | phosphatidylinositol-3,4,5-trisphosphate 5-phosphatase 1 | 28 | 4 | - | - | - | - |  | - | - | - | - | - | - |  | 1 | 0 |
| gi|148225388 | tubulin, alpha 4a | 45 | 3 | - | - | - | - |  | - | - | - | - | - | - |  | 1 | 0 |
| gi|148226581 | ATP-dependent RNA helicase DHX29 | - | - | 35 | 6 | - | - |  | - | - | - | - | - | - |  | 1 | 0 |
| gi|148228823 | uncharacterized protein LOC432274 | - | - | - | - | 35 | 3 |  | - | - | - | - | - | - |  | 1 | 0 |
| gi|148229111 | cytosolic 10-formyltetrahydrofolate dehydrogenase | - | - | 35 | 3 | - | - |  | - | - | - | - | - | - |  | 1 | 0 |
| gi|148229232 | uncharacterized protein LOC379512 precursor | 29 | 1 | - | - | - | - |  | - | - | - | - | - | - |  | 1 | 0 |
| gi|148229939 | glyceraldehyde-3-phosphate dehydrogenase | - | - | - | - | 49 | 4 |  | - | - | - | - | - | - |  | 1 | 0 |
| gi|148230434 | tyrosine kinase 2 | - | - | - | - | 35 | 3 |  | - | - | - | - | - | - |  | 1 | 0 |
| gi|148230436 | phosphoglycerate kinase 1 | 31 | 3 | - | - | - | - |  | - | - | - | - | - | - |  | 1 | 0 |
| gi|148230755 | cordon-bleu homolog | 30 | 3 | - | - | - | - |  | - | - | - | - | - | - |  | 1 | 0 |
| gi|148231041 | phosphoglucomutase 1 | 39 | 4 | - | - | - | - |  | - | - | - | - | - | - |  | 1 | 0 |
| gi|148231603 | lethal giant larvae | - | - | 29 | 5 | - | - |  | - | - | - | - | - | - |  | 1 | 0 |
| gi|148231865 | aldehyde dehydrogenase 1A3 | - | - | 87 | 5 | - | - |  | - | - | - | - | - | - |  | 1 | 0 |
| gi|148232082 | cofilin-1-A | - | - | - | - | 69 | 4 |  | - | - | - | - | - | - |  | 1 | 0 |
| gi|148233390 | histone-lysine N-methyltransferase SUV420H2 | 34 | 6 | - | - | - | - |  | - | - | - | - | - | - |  | 1 | 0 |
| gi|148233902 | methylenetetrahydrofolate dehydrogenase (NADP+ dependent) 1 | 30 | 1 | - | - | - | - |  | - | - | - | - | - | - |  | 1 | 0 |
| gi|148234122 | leukotriene A4 hydrolase | - | - | - | - | 50 | 3 |  | - | - | - | - | - | - |  | 1 | 0 |
| gi|148236559 | ST8 alpha-N-acetyl-neuraminide alpha-2,8-sialyltransferase 2 precursor | 34 | 6 | - | - | - | - |  | - | - | - | - | - | - |  | 1 | 0 |
| gi|148236561 | BTB (POZ) domain containing 8 | - | - | 34 | 8 | - | - |  | - | - | - | - | - | - |  | 1 | 0 |
| gi|148237026 | tryptophan 5-hydroxylase | - | - | - | - | 29 | 3 |  | - | - | - | - | - | - |  | 1 | 0 |
| gi|148237362 | uncharacterized protein LOC414540 | 30 | 3 | - | - | - | - |  | - | - | - | - | - | - |  | 1 | 0 |
| gi|148237614 | plastin 3 | - | - | 35 | 4 | - | - |  | - | - | - | - | - | - |  | 1 | 0 |
| gi|148237794 | ribosomal protein S5 | - | - | - | - | 35 | 2 |  | - | - | - | - | - | - |  | 1 | 0 |
| gi|148922162 | LOC100101335 protein | - | - | 28 | 3 | - | - |  | - | - | - | - | - | - |  | 1 | 0 |
| gi|163915549 | Unknown (protein for IMAGE:7008158) | 204 | 9 | - | - | - | - |  | - | - | - | - | - | - |  | 1 | 0 |
| gi|189217730 | uncharacterized protein KIAA1841 homolog | 28 | 3 | - | - | - | - |  | - | - | - | - | - | - |  | 1 | 0 |
| gi|205360872 | uncharacterized protein LOC100189571 precursor | - | - | - | - | 87 | 5 |  | - | - | - | - | - | - |  | 1 | 0 |
| gi|205360874 | hydroxysteroid (17-beta) dehydrogenase 14 | 36 | 2 | - | - | - | - |  | - | - | - | - | - | - |  | 1 | 0 |
| gi|213626249 | Unknown (protein for MGC:196855) | 32 | 7 | - | - | - | - |  | - | - | - | - | - | - |  | 1 | 0 |
| gi|214113 | elongation factor-1 alpha-chain protein (EF-1-alpha) | - | - | - | - | 137 | 6 |  | - | - | - | - | - | - |  | 1 | 0 |
| gi|257051069 | transitional endoplasmic reticulum ATPase | 49 | 3 | - | - | - | - |  | - | - | - | - | - | - |  | 1 | 0 |
| gi|27695247 | PCK2 protein | - | - | - | - | 29 | 2 |  | - | - | - | - | - | - |  | 1 | 0 |
| gi|28175306 | Tf-b protein | 38 | 1 | - | - | - | - |  | - | - | - | - | - | - |  | 1 | 0 |
| gi|32450649 | VTI1A protein | - | - | 32 | 2 | - | - |  | - | - | - | - | - | - |  | 1 | 0 |
| gi|326322080 | HJURP | - | - | - | - | 42 | 3 |  | - | - | - | - | - | - |  | 1 | 0 |
| gi|350538223 | smooth muscle alpha actin | - | - | - | - | 78 | 9 |  | - | - | - | - | - | - |  | 1 | 0 |
| gi|37589398 | Ube2z protein | 27 | 3 | - | - | - | - |  | - | - | - | - | - | - |  | 1 | 0 |
| gi|40286642 | nucleoporin Nup88B | - | - | - | - | 28 | 4 |  | - | - | - | - | - | - |  | 1 | 0 |
| gi|50414532 | LOC445846 protein | - | - | 35 | 2 | - | - |  | - | - | - | - | - | - |  | 1 | 0 |
| gi|50414757 | LOC445881 protein | - | - | - | - | 32 | 2 |  | - | - | - | - | - | - |  | 1 | 0 |
| gi|51261615 | Unknown (protein for MGC:80766) | 34 | 4 | - | - | - | - |  | - | - | - | - | - | - |  | 1 | 0 |
| gi|54261597 | LOC495281 protein | - | - | - | - | 27 | 4 |  | - | - | - | - | - | - |  | 1 | 0 |
| gi|62740093 | LOC733207 protein | - | - | - | - | 27 | 2 |  | - | - | - | - | - | - |  | 1 | 0 |
| gi|6630877 | fatvg | 42 | 3 | - | - | - | - |  | - | - | - | - | - | - |  | 1 | 0 |
| gi|6636413 | ataxia telangiectasia mutated | 31 | 4 | - | - | - | - |  | - | - | - | - | - | - |  | 1 | 0 |
| gi|6689545 | FCP1 serine phosphatase | - | - | - | - | 31 | 4 |  | - | - | - | - | - | - |  | 1 | 0 |
| gi|76779741 | LOC733355 protein | - | - | - | - | 28 | 5 |  | - | - | - | - | - | - |  | 1 | 0 |
| gi|89275119 | SP22 | - | - | - | - | 30 | 3 |  | - | - | - | - | - | - |  | 1 | 0 |
| gi|147898439 | uncharacterized protein LOC432110 | - | - | - | - | - | - |  | 32 | 2 | - | - | - | - |  | 0 | 1 |
| gi|147899181 | inositol monophosphatase 3 | - | - | - | - | - | - |  | - | - | 29 | 2 | - | - |  | 0 | 1 |
| gi|147899609 | putative sodium-coupled neutral amino acid transporter 10 | - | - | - | - | - | - |  | 31 | 6 | - | - | - | - |  | 0 | 1 |
| gi|147903117 | apoptotic chromatin condensation inducer 1 | - | - | - | - | - | - |  | - | - | 32 | 4 | - | - |  | 0 | 1 |
| gi|148221985 | vimentin-4 | - | - | - | - | - | - |  | - | - | 51 | 5 | - | - |  | 0 | 1 |
| gi|148222597 | heat shock 70kDa protein 1-like | - | - | - | - | - | - |  | 30 | 4 | - | - | - | - |  | 0 | 1 |
| gi|148223409 | uncharacterized protein LOC780761 | - | - | - | - | - | - |  | 100 | 7 | - | - | - | - |  | 0 | 1 |
| gi|148223784 | THO complex subunit 7 homolog | - | - | - | - | - | - |  | 28 | 1 | - | - | - | - |  | 0 | 1 |
| gi|148224397 | tubulin, beta 4A class IVa | - | - | - | - | - | - |  | 37 | 3 | - | - | - | - |  | 0 | 1 |
| gi|148225977 | prostaglandin reductase 1, gene 1 | - | - | - | - | - | - |  | 29 | 2 | - | - | - | - |  | 0 | 1 |
| gi|148226448 | PR domain containing 2, with ZNF domain | - | - | - | - | - | - |  | 29 | 2 | - | - | - | - |  | 0 | 1 |
| gi|148227234 | CDK5 regulatory subunit associated protein 3 | - | - | - | - | - | - |  | 28 | 3 | - | - | - | - |  | 0 | 1 |
| gi|148230965 | trafficking protein particle complex subunit 9 | - | - | - | - | - | - |  | 27 | 2 | - | - | - | - |  | 0 | 1 |
| gi|148231277 | annexin A4 | - | - | - | - | - | - |  | - | - | 257 | 13 | - | - |  | 0 | 1 |
| gi|148234100 | XPA binding protein 2 | - | - | - | - | - | - |  | 29 | 4 | - | - | - | - |  | 0 | 1 |
| gi|148234170 | uncharacterized protein LOC494720 | - | - | - | - | - | - |  | 35 | 2 | - | - | - | - |  | 0 | 1 |
| gi|148235126 | keratin, type II cytoskeletal 8 | - | - | - | - | - | - |  | 67 | 4 | - | - | - | - |  | 0 | 1 |
| gi|148236301 | telomeric repeat binding factor 2 | - | - | - | - | - | - |  | - | - | 28 | 2 | - | - |  | 0 | 1 |
| gi|148236655 | HMG box domain containing 3 | - | - | - | - | - | - |  | - | - | 27 | 3 | - | - |  | 0 | 1 |
| gi|148237263 | serum albumin A precursor | - | - | - | - | - | - |  | 71 | 5 | - | - | - | - |  | 0 | 1 |
| gi|148237741 | thioredoxin | - | - | - | - | - | - |  | 32 | 2 | - | - | - | - |  | 0 | 1 |
| gi|148238241 | abhydrolase domain containing 14B | - | - | - | - | - | - |  | 34 | 2 | - | - | - | - |  | 0 | 1 |
| gi|171460968 | uncharacterized protein LOC431965 | - | - | - | - | - | - |  | - | - | 28 | 2 | - | - |  | 0 | 1 |
| gi|205360870 | histone cluster 1, H4d | - | - | - | - | - | - |  | 67 | 3 | - | - | - | - |  | 0 | 1 |
| gi|205360894 | antigen identified by monoclonal antibody Ki-67 | - | - | - | - | - | - |  | - | - | 36 | 6 | - | - |  | 0 | 1 |
| gi|2497521 | kinesin-like protein KIF11-A | - | - | - | - | - | - |  | 45 | 6 | - | - | - | - |  | 0 | 1 |
| gi|297206779 | heat shock 70 kDa protein 5b precursor | - | - | - | - | - | - |  | - | - | 76 | 5 | - | - |  | 0 | 1 |
| gi|47123069 | LOC431965 protein | - | - | - | - | - | - |  | 33 | 2 | - | - | - | - |  | 0 | 1 |
| gi|48734642 | Unknown (protein for MGC:82324) | - | - | - | - | - | - |  | - | - | 34 | 1 | - | - |  | 0 | 1 |
| gi|4902905 | unnamed protein product | - | - | - | - | - | - |  | 100 | 7 | - | - | - | - |  | 0 | 1 |
| gi|49522287 | LOC443571 protein | - | - | - | - | - | - |  | - | - | 35 | 2 | - | - |  | 0 | 1 |
| gi|50416360 | Pik4ca-prov protein | - | - | - | - | - | - |  | - | - | 30 | 6 | - | - |  | 0 | 1 |
| gi|82132322 | DNA topoisomerase 2-binding protein 1-B | - | - | - | - | - | - |  | 29 | 13 | - | - | - | - |  | 0 | 1 |
| gi|122936451 | LOC494754 protein | - | - | - | - | - | - |  | - | - | - | - | 29 | 4 |  | 0 | 1 |
| gi|1334690 | pro-TGF-beta2 | - | - | - | - | - | - |  | - | - | - | - | 27 | 2 |  | 0 | 1 |
| gi|148222868 | uncharacterized protein LOC379356 | - | - | - | - | - | - |  | - | - | - | - | 27 | 1 |  | 0 | 1 |
| gi|148224696 | YTH domain containing 1 | - | - | - | - | - | - |  | - | - | - | - | 30 | 3 |  | 0 | 1 |
| gi|148232892 | uncharacterized protein C17orf85 homolog | - | - | - | - | - | - |  | - | - | - | - | 28 | 4 |  | 0 | 1 |
| gi|161612103 | Unknown (protein for MGC:181785) | - | - | - | - | - | - |  | - | - | - | - | 31 | 3 |  | 0 | 1 |
| gi|213627659 | Ectodermin | - | - | - | - | - | - |  | - | - | - | - | 30 | 4 |  | 0 | 1 |
| gi|288541396 | actin, cytoplasmic type 5 | - | - | - | - | - | - |  | - | - | - | - | 187 | 13 |  | 0 | 1 |
| gi|51968292 | 20S proteasome alpha5 subunit | - | - | - | - | - | - |  | - | - | - | - | 30 | 2 |  | 0 | 1 |
| gi|54038193 | LOC779025 protein | - | - | - | - | - | - |  | - | - | - | - | 41 | 3 |  | 0 | 1 |
| gi|57921050 | LOC733147 protein | - | - | - | - | - | - |  | - | - | - | - | 41 | 4 |  | 0 | 1 |
| gi|7239234 | mRNA capping enzyme | - | - | - | - | - | - |  | - | - | - | - | 27 | 3 |  | 0 | 1 |
| gi|807696 | x-Delta-1 | - | - | - | - | - | - |  | - | - | - | - | 28 | 2 |  | 0 | 1 |

**Table S2. Complete list of proteins identified as valid**

**The 145 valid proteins identified in the *X*. *laevis* liver in the control and cold-exposure groups are shown. The number of detections in triplicate LC-MS/MS runs, Mascot protein scores and peptide contents, fold change in protein abundance (cold exposure/control), and correspondent human homologous proteins are also included. The proteins found to be differentially expressed were categorized into four groups as follow: group 1, proteins up-regulated by cold exposure (fold change > 1.25); group 2, proteins detected only in cold exposure and not in the control (i.e., considered to be newly induced); group 3, proteins down-regulated with cold exposure (fold change < 0.8); and group 4, proteins below the detection limit under cold exposure (i.e., detected only in the control and not in cold exposure).**

| **Accession number** | **Protein name** | **Number of detection** | |  | **Control*^a^*** | |  | **Cold exposure*^a^*** | |  | **Fold change*^b^*** | | |  | | **Human homologous protein** | | | |
| --- | --- | --- | --- | --- | --- | --- | --- | --- | --- | --- | --- | --- | --- | --- | --- | --- | --- | --- | --- |
|  |  | **Control** | **Cold exposure** |  | **Protein score** | **Peptide content** |  | **Protein score** | **Peptide content** |  | **Mean** | **CV**  **(%)** | |  | | **RefSeq protein ID*^c^*** | | | **Protein name** |
| ***group 1: Up-regulated proteins in cold-exposed X. laevis liver*** | | | |  |  |  |  |  |  |  |  |  | |  | |  | | |  |
| gi|147906883 | hemoglobin subunit beta-1 | 3 | 3 |  | 421 | 40 |  | 491 | 62 |  | 16.8 | 128 | |  | | NP_000509^†^ | | | hemoglobin subunit beta |
| gi|54037970 | LOC495053 protein | 3 | 2 |  | 41 | 8 |  | 52 | 13 |  | 9.79 | 136 | |  | | NP_002696 | | | periplakin |
| gi|147902603 | hemoglobin subunit alpha-2 | 3 | 3 |  | 252 | 45 |  | 322 | 58 |  | 7.47 | 65.5 | |  | | NP_000508^†^ | | | hemoglobin subunit alpha |
| gi|122285 | hemoglobin subunit alpha-1 | 3 | 3 |  | 338 | 48 |  | 403 | 65 |  | 7.13 | 65.3 | |  | | NP_000508^†^ | | | hemoglobin subunit alpha |
| gi|148223115 | fumarylacetoacetase | 3 | 3 |  | 101 | 7 |  | 121 | 8 |  | 7.01 | 81.7 | |  | | NP_000128 | | | fumarylacetoacetase |
| gi|62740095 | LOC733209 protein | 2 | 2 |  | 35 | 8 |  | 33 | 7 |  | 3.09 | 72.4 | |  | | NP_004478^†^ | | | Golgin subfamily B member 1 isoform 2 |
| gi|291290905 | hemoglobin, gamma G | 3 | 3 |  | 211 | 16 |  | 263 | 24 |  | 2.81 | 18.7 | |  | | NP_000175^†^ | | | hemoglobin subunit gamma-2 |
| gi|118384 | ornithine decarboxylase 1 | 3 | 3 |  | 31 | 3 |  | 38 | 4 |  | 2.67 | 89.1 | |  | | NP_002530 | | | ornithine decarboxylase |
| gi|147906522 | potassium voltage-gated channel, Shab-related subfamily, member 2 | 3 | 2 |  | 39 | 8 |  | 38 | 12 |  | 2.64 | 45.9 | |  | | NP_004761 | | | potassium voltage-gated channel subfamily B member 2 |
| gi|147898869 | purine nucleoside phosphorylase | 3 | 2 |  | 90 | 7 |  | 84 | 5 |  | 1.72 | 18.5 | |  | | NP_000261 | | | purine nucleoside phosphorylase |
| gi|148222055 | similar to carbonic anhydrase II | 2 | 3 |  | 54 | 2 |  | 57 | 1 |  | 1.48 | 22.2 | |  | | NP_940986 | | | carbonic anhydrase 13 |
| gi|147899037 | malate dehydrogenase 2, NAD (mitochondrial) | 2 | 3 |  | 55 | 5 |  | 96 | 5 |  | 1.37 | 27.6 | |  | | NP_005909 | | | malate dehydrogenase, mitochondrial precursor |
| gi|147904511 | enolase 3 | 2 | 2 |  | 66 | 8 |  | 72 | 7 |  | 1.37 | 32.4 | |  | | NP_001967 | | | beta-enolase isoform 1 |
| gi|11385422 | serine/threonine-protein kinase atr | 2 | 3 |  | 57 | 7 |  | 57 | 5 |  | 1.29 | 17.2 | |  | | NP_001175 | | | serine/threonine-protein kinase ATR |
| ***group 2: Newly induced proteins in cold-exposed X. laevis liver*** | | | |  |  |  |  |  |  |  |  |  | |  | |  | | |  |
| gi|125858908 | Unknown (protein for IMAGE:8550378) | 0 | 2 |  | 0 | 0 |  | 30 | 5 |  |  |  | |  | | NP_005073^†^ | | | E3 ubiquitin/ISG15 ligase TRIM25 |
| gi|147898691 | DNA-dependent protein kinase catalytic subunit | 0 | 2 |  | 0 | 0 |  | 33 | 8 |  |  |  | |  | | NP_008835^†^ | | | DNA-dependent protein kinase catalytic subunit isoform 1 |
| gi|147901600 | liver glycogen phosphorylase | 0 | 2 |  | 0 | 0 |  | 35 | 4 |  |  |  | |  | | NP_002854 | | | glycogen phosphorylase, liver form isoform 1 |
| gi|147902854 | zinc finger protein 507 | 0 | 2 |  | 0 | 0 |  | 31 | 5 |  |  |  | |  | | NP_055725^†^ | | | zinc finger protein 507 |
| gi|147905626 | alcohol dehydrogenase 1C (class I), gamma polypeptide | 0 | 2 |  | 0 | 0 |  | 35 | 2 |  |  |  | |  | | NP_000660 | | | alcohol dehydrogenase 1C |
| gi|147905834 | lysosomal thioesterase PPT2-B precursor | 0 | 2 |  | 0 | 0 |  | 58 | 11 |  |  |  | |  | | NP_005146^†^ | | | lysosomal thioesterase PPT2 isoform a precursor |
| gi|147907415 | ropporin-1-like protein | 0 | 2 |  | 0 | 0 |  | 28 | 3 |  |  |  | |  | | NP_001188395 | | | ropporin-1-like protein |
| gi|148225833 | glycerol-3-phosphate dehydrogenase 1 (soluble) | 0 | 2 |  | 0 | 0 |  | 54 | 3 |  |  |  | |  | | NP_005267 | | | glycerol-3-phosphate dehydrogenase [NAD+], cytoplasmic isoform 1 |
| gi|160420189 | dopa decarboxylase | 0 | 2 |  | 0 | 0 |  | 32 | 6 |  |  |  | |  | | NP_001076440 | | | aromatic-L-amino-acid decarboxylase isoform 1 |
| gi|3745759 | histone H4 | 0 | 2 |  | 0 | 0 |  | 75 | 3 |  |  |  | |  | | NP_778224^†^ | | | histone H4 |
| gi|37791449 | protein tyrosine phosphatase PTP-PEST | 0 | 2 |  | 0 | 0 |  | 37 | 4 |  |  |  | |  | | NP_002826^†^ | | | tyrosine-protein phosphatase non-receptor type 12 isoform 1 |
| gi|49115587 | LOC443650 protein | 0 | 2 |  | 0 | 0 |  | 32 | 3 |  |  |  | |  | | NP_055807 | | | disheveled-associated activator of morphogenesis 1 |
| gi|68534041 | LOC733291 protein | 0 | 2 |  | 0 | 0 |  | 55 | 4 |  |  |  | |  | | NP_006301 | | | puromycin-sensitive aminopeptidase |
| ***group 3: Down-regulated proteins in cold-exposed X. laevis liver*** | | | | | |  |  |  |  |  |  |  | |  | |  | | |  |
| gi|33585701 | MGC69098 protein | 2 | 2 |  | 49 | 3 |  | 43 | 2 |  | 0.631 | 24.3 | |  | | NP_005882 | | | acetyl-CoA acetyltransferase, cytosolic |
| gi|148236249 | prosaposin precursor | 3 | 3 |  | 74 | 6 |  | 56 | 3 |  | 0.677 | 27.5 | |  | | NP_002769^†^ | | | proactivator polypeptide isoform a preproprotein |
| gi|147902842 | annexin A13 | 3 | 2 |  | 75 | 4 |  | 75 | 3 |  | 0.693 | 22.2 | |  | | NP_001003954 | | | annexin A13 isoform b |
| gi|148233183 | elongation factor 1-alpha, somatic form | 2 | 3 |  | 159 | 8 |  | 127 | 7 |  | 0.728 | 15.2 | |  | | NP_001393^†^ | | | elongation factor 1-alpha 1 |
| gi|148226440 | MGC82879 protein | 3 | 3 |  | 547 | 29 |  | 445 | 24 |  | 0.730 | 20.0 | |  | | NP_001144 | | | annexin A4 |
| gi|113571 | serum albumin B | 3 | 2 |  | 94 | 6 |  | 103 | 5 |  | 0.750 | 17.9 | |  | | NP_000468^†^ | | | serum albumin preproprotein |
| gi|148237649 | sorbitol dehydrogenase | 3 | 2 |  | 76 | 8 |  | 41 | 4 |  | 0.750 | 34.6 | |  | | NP_003095 | | | sorbitol dehydrogenase |
| gi|16332351 | glutathione S-transferase mu 2 | 3 | 3 |  | 466 | 21 |  | 360 | 16 |  | 0.750 | 18.0 | |  | | NP_000839 | | | glutathione S-transferase Mu 2 isoform 1 |
| gi|148222492 | uncharacterized protein LOC495316 | 3 | 2 |  | 203 | 11 |  | 96 | 8 |  | 0.761 | 16.3 | |  | | NP_001087 | | | ATP-citrate synthase isoform 1 |
| ***group 4: Proteins below detection limit in cold-exposed X. laevis liver*** | | | | | |  |  |  |  |  |  |  | |  | |  | | |  |
| gi|147902026 | peroxiredoxin 6 | 3 | 0 |  | 38 | 4 |  | 0 | 0 |  |  |  | |  | | NP_004896 | | | peroxiredoxin-6 |
| gi|148232992 | enolase 1 a | 3 | 0 |  | 105 | 9 |  | 0 | 0 |  |  |  | |  | | NP_001419 | | | alpha-enolase isoform 1 |
| gi|50417653 | LOC397850 protein | 3 | 0 |  | 75 | 3 |  | 0 | 0 |  |  |  | |  | | NP_005338 | | | 78 kDa glucose-regulated protein precursor |
| gi|64659 | elongation factor 1-alpha | 3 | 0 |  | 47 | 3 |  | 0 | 0 |  |  |  | |  | | NP_001393 | | | elongation factor 1-alpha 1 |
| gi|11493740 | DNA polymerase epsilon | 2 | 0 |  | 50 | 9 |  | 0 | 0 |  |  |  | |  | | NP_006222 | | | DNA polymerase epsilon catalytic subunit A |
| gi|147899332 | glucan (1,4-alpha-), branching enzyme 1 | 2 | 0 |  | 63 | 8 |  | 0 | 0 |  |  |  | |  | | NP_000149 | | | 1,4-alpha-glucan-branching enzyme |
| gi|147902366 | cathepsin C precursor | 2 | 0 |  | 39 | 4 |  | 0 | 0 |  |  |  | |  | | NP_001805 | | | dipeptidyl peptidase 1 isoform a preproprotein |
| gi|147904142 | topoisomerase (DNA) II binding protein 1 | 2 | 0 |  | 31 | 15 |  | 0 | 0 |  |  |  | |  | | NP_008958 | | | DNA topoisomerase 2-binding protein 1 |
| gi|147905009 | pericentriolar material 1 protein | 2 | 0 |  | 38 | 9 |  | 0 | 0 |  |  |  | |  | | NP_006188^†^ | | | pericentriolar material 1 protein |
| gi|147906799 | lethal giant larvae homolog 1 | 2 | 0 |  | 36 | 6 |  | 0 | 0 |  |  |  | |  | | NP_004131 | | | lethal(2) giant larvae protein homolog 1 |
| gi|147906817 | tumor necrosis factor, alpha-induced protein 3 | 2 | 0 |  | 33 | 4 |  | 0 | 0 |  |  |  | |  | | NP_006281^†^ | | | tumor necrosis factor alpha-induced protein 3 |
| gi|148222987 | glucoside xylosyltransferase 2 precursor | 2 | 0 |  | 35 | 1 |  | 0 | 0 |  |  |  | |  | | NP_001073862 | | | glucoside xylosyltransferase 2 precursor |
| gi|148229521 | protein CIP2A homolog | 2 | 0 |  | 32 | 5 |  | 0 | 0 |  |  |  | |  | | NP_065941 | | | protein CIP2A |
| gi|148233056 | pyruvate kinase, liver and RBC | 2 | 0 |  | 46 | 2 |  | 0 | 0 |  |  |  | |  | | NP_002645 | | | pyruvate kinase isozymes M1/M2 isoform a |
| gi|148235435 | uncharacterized protein LOC398814 | 2 | 0 |  | 99 | 6 |  | 0 | 0 |  |  |  | |  | | NP_006750 | | | UTP--glucose-1-phosphate uridylyltransferase isoform a |
| gi|148237900 | adenosylhomocysteinase B | 2 | 0 |  | 82 | 5 |  | 0 | 0 |  |  |  | |  | | NP_000678 | | | adenosylhomocysteinase isoform 1 |
| gi|159155766 | LOC780759 protein | 2 | 0 |  | 69 | 6 |  | 0 | 0 |  |  |  | |  | | NP_001128527 | | | transketolase |
| gi|18202614 | dimerization cofactor of hepatocyte nuclear factor 1-alpha | 2 | 0 |  | 48 | 2 |  | 0 | 0 |  |  |  | |  | | NP_000272 | | | pterin-4-alpha-carbinolamine dehydratase precursor |
| gi|288557262 | heat shock cognate 70.II protein | 2 | 0 |  | 71 | 5 |  | 0 | 0 |  |  |  | |  | | NP_006588 | | | heat shock cognate 71 kDa protein isoform 1 |
| gi|50415517 | heat shock 70 kDa protein | 2 | 0 |  | 78 | 3 |  | 0 | 0 |  |  |  | |  | | NP_006588 | | | heat shock cognate 71 kDa protein isoform 1 |
| gi|51703567 | MGC84331 protein | 2 | 0 |  | 31 | 7 |  | 0 | 0 |  |  |  | |  | | NP_078857 | | | protein FAM184A isoform 1 |
| gi|71051388 | matrix metalloproteinase-18 precursor | 2 | 0 |  | 38 | 3 |  | 0 | 0 |  |  |  | |  | | NP_002412^†^ | | | interstitial collagenase isoform 1 preproprotein [Homo sapiens] |
| ***Not differentially expressed*** | |  |  |  |  |  |  |  |  |  |  |  |  | |  | | |  | |
| gi|120577551 | glyoxylate reductase/hydroxypyruvate reductase-like protein | 2 | 3 |  | 163 | 11 |  | 146 | 8 |  | 0.807 | 23.7 | |  | | | NP_036335^†^ | | glyoxylate reductase/hydroxypyruvate reductase |
| gi|148231271 | aldehyde dehydrogenase 9 family, member A1 | 3 | 3 |  | 553 | 29 |  | 468 | 24 |  | 0.813 | 17.0 | |  | | | NP_000687 | | 4-trimethylaminobutyraldehyde dehydrogenase |
| gi|148230001 | GTP cyclohydrolase 1 feedback regulatory protein | 3 | 2 |  | 53 | 2 |  | 54 | 3 |  | 0.818 | 19.5 | |  | | | NP_005249 | | GTP cyclohydrolase 1 feedback regulatory protein |
| gi|148233713 | arginase 1 | 3 | 3 |  | 414 | 20 |  | 374 | 18 |  | 0.819 | 18.4 | |  | | | NP_000036 | | arginase-1 isoform 2 |
| gi|32450751 | LOC398623 protein | 3 | 3 |  | 290 | 14 |  | 272 | 13 |  | 0.821 | 21.2 | |  | | | NP_000026 | | fructose-bisphosphate aldolase B |
| gi|1065161 | superoxide dismutase [Cu-Zn] B | 3 | 2 |  | 171 | 5 |  | 139 | 5 |  | 0.821 | 20.1 | |  | | | NP_000445^†^ | | superoxide dismutase [Cu-Zn] |
| gi|148232311 | nucleolar and spindle-associated protein 1-A | 3 | 2 |  | 34 | 3 |  | 29 | 1 |  | 0.823 | 19.7 | |  | | | NP_060924^†^ | | nucleolar and spindle-associated protein 1 isoform 2 |
| gi|148227690 | lactate dehydrogenase A | 3 | 3 |  | 287 | 15 |  | 285 | 13 |  | 0.823 | 19.2 | |  | | | NP_001158886 | | L-lactate dehydrogenase A chain isoform 3 |
| gi|64647 | Cu-Zn superoxide dismutase C-terminal fragment | 3 | 3 |  | 128 | 4 |  | 94 | 6 |  | 0.825 | 22.7 | |  | | | NP_000445 | | superoxide dismutase [Cu-Zn] |
| gi|148223127 | mg:bb02e05 | 3 | 3 |  | 195 | 11 |  | 176 | 10 |  | 0.839 | 18.9 | |  | | | NP_002037 | | glyceraldehyde-3-phosphate dehydrogenase isoform 1 |
| gi|1703127 | actin, cytoplasmic type 8 | 3 | 2 |  | 237 | 16 |  | 182 | 12 |  | 0.849 | 22.3 | |  | | | NP_001077007 | | POTE ankyrin domain family member E |
| gi|148232264 | argininosuccinate lyase | 3 | 3 |  | 267 | 13 |  | 154 | 8 |  | 0.851 | 17.4 | |  | | | NP_000039 | | argininosuccinate lyase isoform 1 |
| gi|33416812 | lymphocyte cytosolic protein 1 (L-plastin) | 2 | 2 |  | 48 | 3 |  | 64 | 4 |  | 0.873 | 15.1 | |  | | | NP_002289 | | plastin-2 |
| gi|118136396 | catalase, gene 2 | 3 | 3 |  | 498 | 31 |  | 490 | 27 |  | 0.875 | 19.2 | |  | | | NP_001743 | | catalase |
| gi|147898737 | transketolase | 3 | 3 |  | 110 | 7 |  | 70 | 5 |  | 0.877 | 18.9 | |  | | | NP_001128527 | | transketolase |
| gi|148223641 | catalase, gene 2 | 2 | 3 |  | 495 | 31 |  | 480 | 25 |  | 0.881 | 20.9 | |  | | | NP_001743 | | catalase |
| gi|148234947 | UDP-glucose pyrophosphorylase 2 | 3 | 3 |  | 202 | 11 |  | 216 | 11 |  | 0.894 | 17.3 | |  | | | NP_006750 | | UTP--glucose-1-phosphate uridylyltransferase isoform a |
| gi|147902599 | uncharacterized protein LOC398893 | 3 | 3 |  | 158 | 6 |  | 113 | 4 |  | 0.897 | 17.2 | |  | | | NP_002141 | | 4-hydroxyphenylpyruvate dioxygenase isoform 1 |
| gi|9910617 | allantoicase | 3 | 3 |  | 89 | 6 |  | 110 | 8 |  | 0.903 | 21.3 | |  | | | NP_060906 | | probable allantoicase isoform a |
| gi|147899575 | prostaglandin D2 synthase, hematopoietic a | 3 | 3 |  | 332 | 17 |  | 315 | 16 |  | 0.904 | 19.7 | |  | | | NP_055300^†^ | | hematopoietic prostaglandin D synthase |
| gi|147907284 | betaine--homocysteine S-methyltransferase 1 | 3 | 3 |  | 297 | 20 |  | 230 | 16 |  | 0.908 | 18.8 | |  | | | NP_001704 | | betaine--homocysteine S-methyltransferase 1 |
| gi|55824753 | LOC495840 protein | 3 | 3 |  | 175 | 13 |  | 138 | 11 |  | 0.911 | 16.0 | |  | | | NP_001743 | | catalase |
| gi|147904364 | epoxide hydrolase 2, cytoplasmic | 2 | 2 |  | 45 | 8 |  | 51 | 6 |  | 0.914 | 30.3 | |  | | | NP_001970^†^ | | epoxide hydrolase 2 isoform a |
| gi|21952442 | prostaglandin D2 synthase, hematopoietic b | 3 | 3 |  | 147 | 8 |  | 146 | 7 |  | 0.918 | 17.5 | |  | | | NP_055300^†^ | | hematopoietic prostaglandin D synthase |
| gi|10197483 | aldolase B, fructose-bisphosphate | 3 | 3 |  | 293 | 14 |  | 275 | 12 |  | 0.930 | 19.2 | |  | | | NP_000026 | | fructose-bisphosphate aldolase B |
| gi|148237546 | MGC83376 protein | 3 | 3 |  | 313 | 20 |  | 189 | 14 |  | 0.931 | 18.7 | |  | | | NP_000660 | | alcohol dehydrogenase 1C |
| gi|147905276 | transaldolase 1 | 3 | 3 |  | 153 | 9 |  | 83 | 6 |  | 0.940 | 23.1 | |  | | | NP_006746 | | transaldolase |
| gi|148229158 | transketolase-like 2 | 3 | 3 |  | 152 | 9 |  | 129 | 12 |  | 0.943 | 19.8 | |  | | | NP_115512 | | transketolase-like protein 2 |
| gi|147900682 | selenium-binding protein 1-B | 3 | 3 |  | 119 | 9 |  | 98 | 7 |  | 0.944 | 30.5 | |  | | | NP_003935 | | selenium-binding protein 1 |
| gi|148234425 | glucose-6-phosphate isomerase | 3 | 3 |  | 91 | 7 |  | 49 | 5 |  | 0.965 | 19.8 | |  | | | NP_000166 | | glucose-6-phosphate isomerase isoform 2 |
| gi|148232692 | MGC80314 protein | 2 | 3 |  | 48 | 2 |  | 61 | 2 |  | 0.966 | 22.3 | |  | | | NP_003320 | | thioredoxin isoform 1 |
| gi|147907224 | 6-phosphogluconate dehydrogenase, decarboxylating | 3 | 3 |  | 124 | 5 |  | 70 | 4 |  | 0.966 | 18.8 | |  | | | NP_002622 | | 6-phosphogluconate dehydrogenase, decarboxylating |
| gi|148236059 | aldo-keto reductase family 1, member C1 | 2 | 3 |  | 44 | 4 |  | 56 | 3 |  | 0.994 | 13.0 | |  | | | NP_003730 | | aldo-keto reductase family 1 member C3 isoform 1 |
| gi|11035016 | SWI/SNF related, matrix associated, actin dependent regulator of chromatin, subfamily a, member 5 | 2 | 3 |  | 54 | 8 |  | 38 | 8 |  | 1.00 | 17.3 | |  | | | NP_003592 | | SWI/SNF related, matrix associated, actin dependent regulator of chromatin, subfamily a, member 5 |
| gi|148230659 | glutamate dehydrogenase 1 | 3 | 3 |  | 124 | 7 |  | 72 | 5 |  | 1.01 | 28.5 | |  | | | NP_005262 | | glutamate dehydrogenase 1, mitochondrial precursor |
| gi|147906867 | eukaryotic translation elongation factor 2, gene 1 | 2 | 2 |  | 51 | 6 |  | 51 | 3 |  | 1.01 | 1.51 | |  | | | NP_001952 | | elongation factor 2 |
| gi|148230238 | homogentisate 1,2-dioxygenase | 3 | 3 |  | 94 | 6 |  | 101 | 7 |  | 1.02 | 25.4 | |  | | | NP_000178 | | homogentisate 1,2-dioxygenase |
| gi|32450739 | alanine-glyoxylate aminotransferase | 3 | 2 |  | 113 | 8 |  | 43 | 3 |  | 1.03 | 25.5 | |  | | | NP_000021 | | serine--pyruvate aminotransferase |
| gi|147902535 | MGC83388 protein | 3 | 3 |  | 61 | 2 |  | 56 | 2 |  | 1.03 | 17.2 | |  | | | NP_004332^†^ | | CAD protein |
| gi|148234619 | carbamoyl-phosphate synthase 1, mitochondrial | 3 | 3 |  | 366 | 27 |  | 410 | 21 |  | 1.04 | 20.6 | |  | | | NP_001116105 | | carbamoyl-phosphate synthase [ammonia], mitochondrial isoform a precursor |
| gi|7416136 | regucalcin (senescence marker protein-30) | 2 | 3 |  | 109 | 8 |  | 75 | 6 |  | 1.05 | 18.3 | |  | | | NP_004674 | | regucalcin |
| gi|148223868 | malate dehydrogenase, cytoplasmic | 2 | 3 |  | 48 | 2 |  | 44 | 2 |  | 1.06 | 17.7 | |  | | | NP_005908 | | malate dehydrogenase, cytoplasmic isoform 2 |
| gi|148228255 | aldehyde dehydrogenase 1 family, member A1 | 3 | 3 |  | 390 | 21 |  | 289 | 19 |  | 1.07 | 18.4 | |  | | | NP_000680 | | retinal dehydrogenase 1 |
| gi|148236091 | fructose-1,6-bisphosphatase 1 | 3 | 3 |  | 62 | 4 |  | 102 | 4 |  | 1.08 | 18.8 | |  | | | NP_001121100 | | fructose-1,6-bisphosphatase 1 |
| gi|112688 | 14-3-3-like protein | 3 | 3 |  | 100 | 9 |  | 110 | 8 |  | 1.10 | 18.2 | |  | | | NP_647539 | | 14-3-3 protein beta/alpha |
| gi|147898618 | L-lactate dehydrogenase A chain | 3 | 3 |  | 91 | 3 |  | 98 | 4 |  | 1.10 | 17.4 | |  | | | NP_005557 | | L-lactate dehydrogenase A chain isoform 1 |
| gi|148235865 | cold-inducible RNA-binding protein B | 3 | 3 |  | 50 | 4 |  | 58 | 3 |  | 1.10 | 31.7 | |  | | | NP_001271 | | cold-inducible RNA-binding protein |
| gi|148226821 | cofilin-1-B | 2 | 3 |  | 66 | 2 |  | 77 | 3 |  | 1.11 | 23.0 | |  | | | NP_005498 | | cofilin-1 |
| gi|148229471 | glucose-6-phosphate dehydrogenase | 3 | 3 |  | 53 | 5 |  | 102 | 4 |  | 1.11 | 29.1 | |  | | | NP_000393 | | glucose-6-phosphate 1-dehydrogenase isoform a |
| gi|148229659 | uncharacterized protein LOC379555 | 3 | 3 |  | 200 | 10 |  | 244 | 9 |  | 1.12 | 17.7 | |  | | | NP_006160† | | nicotinamide N-methyltransferase |
| gi|148224415 | L-lactate dehydrogenase B chain | 3 | 3 |  | 112 | 6 |  | 115 | 7 |  | 1.12 | 21.4 | |  | | | NP_001167568 | | L-lactate dehydrogenase B chain |
| gi|6225751 | nucleoside diphosphate kinase A1 | 3 | 2 |  | 66 | 4 |  | 82 | 4 |  | 1.15 | 20.4 | |  | | | NP_001018146 | | NME1-NME2 protein |
| gi|4586546 | aldehyde dehydrogenase 1A | 3 | 3 |  | 377 | 23 |  | 292 | 18 |  | 1.19 | 26.8 | |  | | | NP_000680 | | retinal dehydrogenase 1 |
| gi|148232240 | isocitrate dehydrogenase 1 | 3 | 3 |  | 73 | 6 |  | 66 | 7 |  | 1.20 | 26.4 | |  | | | NP_005887 | | isocitrate dehydrogenase [NADP] cytoplasmic |
| gi|308153262 | family with sequence similarity 64, member A | 3 | 2 |  | 38 | 3 |  | 41 | 3 |  | 1.21 | 11.7 | |  | | | NP_061886^†^ | | protein FAM64A isoform 1 |
| gi|147900590 | argininosuccinate synthase | 3 | 3 |  | 450 | 30 |  | 431 | 27 |  | 1.21 | 39.0 | |  | | | NP_000041 | | argininosuccinate synthase |
| gi|148236351 | triosephosphate isomerase | 3 | 3 |  | 109 | 5 |  | 119 | 4 |  | 1.23 | 26.8 | |  | | | NP_001152759 | | triosephosphate isomerase isoform 2 |
| gi|77748240 | MGC82659 protein | 3 | 3 |  | 173 | 6 |  | 184 | 6 |  | 1.23 | 17.8 | |  | | | NP_002558 | | phosphatidylethanolamine-binding protein 1 preproprotein |
| ***Detected in control with valid*** | |  |  |  |  |  |  |  |  |  |  |  |  | |  | | |  | |
| gi|147903225 | glutamic-oxaloacetic transaminase 1, soluble | 3 | 1 |  | 65 | 3 |  | 34 | 1 |  |  |  | |  | | | NP_002070 | | aspartate aminotransferase, cytoplasmic |
| gi|147903369 | ribosomal protein L6 | 3 | 1 |  | 40 | 2 |  | 30 | 1 |  |  |  | |  | | | NP_000961 | | 60S ribosomal protein L6 |
| gi|147906560 | male-specific lethal 3 homolog | 3 | 1 |  | 32 | 3 |  | 32 | 2 |  |  |  | |  | | | NP_523353 | | male-specific lethal 3 homolog isoform a |
| gi|148222521 | ATP citrate lyase | 3 | 1 |  | 166 | 10 |  | 75 | 8 |  |  |  | |  | | | NP_001087 | | ATP-citrate synthase isoform 1 |
| gi|148225538 | tyrosine 3-monooxygenase/tryptophan 5-monooxygenase activation protein, epsilon polypeptide | 3 | 1 |  | 98 | 8 |  | 53 | 3 |  |  |  | |  | | | NP_006752 | | 14-3-3 protein epsilon |
| gi|148227210 | zinc finger protein 367 | 3 | 1 |  | 43 | 3 |  | 43 | 2 |  |  |  | |  | | | NP_710162 | | zinc finger protein 367 |
| gi|148234370 | peroxiredoxin 5 precursor | 3 | 1 |  | 54 | 4 |  | 43 | 2 |  |  |  | |  | | | NP_036226 | | peroxiredoxin-5, mitochondrial isoform a precursor |
| gi|148235689 | alpha-enolase | 3 | 1 |  | 157 | 11 |  | 75 | 6 |  |  |  | |  | | | NP_001419 | | alpha-enolase isoform 1 |
| gi|171846437 | LOC100158433 protein | 3 | 1 |  | 31 | 7 |  | 28 | 4 |  |  |  | |  | | | NP_005471^†^ | | tastin isoform 1 |
| gi|27882192 | glyceraldehyde-3-phosphate dehydrogenase | 3 | 1 |  | 139 | 7 |  | 63 | 5 |  |  |  | |  | | | NP_002037 | | glyceraldehyde-3-phosphate dehydrogenase isoform 1 |
| gi|147902134 | aldehyde dehydrogenase 1 family, member A2 | 2 | 1 |  | 53 | 3 |  | 30 | 2 |  |  |  | |  | | | NP_003879 | | retinal dehydrogenase 2 isoform 1 |
| gi|147904296 | uncharacterized protein LOC494651 | 2 | 1 |  | 31 | 3 |  | 30 | 2 |  |  |  | |  | | | NP_055271 | | programmed cell death protein 4 isoform 1 |
| gi|148223708 | zinc finger protein 703-B | 2 | 1 |  | 30 | 1 |  | 32 | 2 |  |  |  | |  | | | NP_079345 | | zinc finger protein 703 |
| gi|148224943 | selenium-binding protein 1-A | 2 | 1 |  | 66 | 6 |  | 48 | 4 |  |  |  | |  | | | NP_003935 | | selenium-binding protein 1 |
| gi|148232365 | structural maintenance of chromosomes 1A | 2 | 1 |  | 37 | 5 |  | 28 | 2 |  |  |  | |  | | | NP_006297 | | structural maintenance of chromosomes protein 1A |
| gi|148234205 | aldo-keto reductase family 1, member C-like 1 | 2 | 1 |  | 36 | 5 |  | 46 | 3 |  |  |  | |  | | | NP_697021 | | alcohol dehydrogenase [NADP(+)] |
| gi|156119465 | keratin, type II cytoskeletal | 2 | 1 |  | 69 | 5 |  | 51 | 3 |  |  |  | |  | | | NP_004684^†^ | | keratin, type II cytoskeletal 75 |
| gi|157426935 | uncharacterized protein LOC100125664 | 2 | 1 |  | 64 | 5 |  | 53 | 5 |  |  |  | |  | | | NP_001743 | | catalase |
| gi|27695014 | MGC64293 protein | 2 | 1 |  | 53 | 2 |  | 45 | 1 |  |  |  | |  | | | NP_005013^†^ | | profilin-1 |
| gi|28436918 | prolyl 4-hydroxylase, beta polypeptide precursor | 2 | 1 |  | 92 | 4 |  | 82 | 6 |  |  |  | |  | | | NP_000909 | | protein disulfide-isomerase precursor |
| gi|33411670 | E3 ubiquitin-protein ligase RNF12-A | 2 | 1 |  | 30 | 1 |  | 31 | 1 |  |  |  | |  | | | NP_057204 | | E3 ubiquitin-protein ligase RLIM |
| gi|46249667 | LOC414586 protein | 2 | 1 |  | 34 | 2 |  | 43 | 2 |  |  |  | |  | | | NP_699160^†^ | | aldehyde dehydrogenase family 16 member A1 isoform 1 |
| gi|7439974 | cold-inducible RNA-binding protein A | 2 | 1 |  | 39 | 3 |  | 39 | 2 |  |  |  | |  | | | NP_001271^†^ | | cold-inducible RNA-binding protein |
| ***Detected in cold-exposed with valid*** | |  |  |  |  |  |  |  |  |  |  |  |  | |  | | |  | |
| gi|148230513 | adenosylhomocysteinase A | 1 | 3 |  | 143 | 5 |  | 106 | 4 |  |  |  | |  | | | NP_000678 | | adenosylhomocysteinase isoform 1 |
| gi|138532 | vimentin-4 | 1 | 2 |  | 29 | 2 |  | 52 | 4 |  |  |  | |  | | | NP_003371 | | vimentin |
| gi|148222316 | uncharacterized protein LOC379202 | 1 | 2 |  | 55 | 3 |  | 42 | 4 |  |  |  | |  | | | NP_006079 | | tubulin beta-4B chain |
| gi|148237590 | malate dehydrogenase 2, NAD (mitochondrial) | 1 | 2 |  | 52 | 5 |  | 104 | 7 |  |  |  | |  | | | NP_005909 | | malate dehydrogenase, mitochondrial precursor |
| gi|32450581 | peptidylprolyl isomerase A (cyclophilin A) | 1 | 2 |  | 32 | 1 |  | 31 | 1 |  |  |  | |  | | | NP_066953 | | peptidyl-prolyl cis-trans isomerase A |
| gi|64580 | beta-2 globin | 1 | 2 |  | 184 | 11 |  | 226 | 21 |  |  |  | |  | | | NP_000509^†^ | | hemoglobin subunit beta |

*^a^* Protein scores and peptide contents in MASCOT MS/MS ion search. Maximum values for triplicate data are shown.

*^b^* Fold changes (cold exposure/control) of protein expression. CV, coefficient of variation.

*^c^* RefSeq IDs of human homologues were obtained from NCBI HomoloGene database or by aligning with NCBI Reference Sequence the (RefSeq) database using the blastp program (^†^).

**Table S3. Significantly enriched Gene Ontology Biological Process (GO:BP) terms found in the control and cold-exposed *X. laevis* livers.**

Enriched analysis for GO:BP terms was performed using the DAVID program with candidate human homologues. False discovery rate (FDR)-corrected *P* values were determined by modified Fisher’s exact test with Benjamini and Hochberg FDR correction. The significantly enriched GO terms (FDR-corrected *P* < 0.01) appearing deepest in the hierarchy were selected. Control- and cold exposure-specific GO:BP terms are shown with FDR-corrected *P* values.

| **GO ID** | **GO:BP Term** | **Count***^a^* | **Proteins (RefSeq Accession, human)** | **FDR-corrected *P* value** |
| --- | --- | --- | --- | --- |
| **Control specifically-enriched** | |  |  |  |
| GO:0051384 | response to glucocorticoid stimulus | 6 | NP_001116105, NP_002070, NP_000026, NP_002558, NP_000021, NP_000039 | 5.39×10^–3^ |
| GO:0034599 | cellular response to oxidative stress | 5 | NP_000393, NP_004896, NP_001743, NP_036226, NP_000445 | 5.61×10^–3^ |
| **Cold-exposure specifically-enriched** | |  |  |  |
| GO:0006734 | NADH metabolic process | 4 | NP_000026, NP_005267, NP_005909, NP_005908 | 0.31×10^–3^ |
| GO:0006094 | Gluconeogenesis | 5 | NP_000026, NP_005267, NP_001152759, NP_001121100, NP_000166 | 0.34×10^–3^ |
| GO:0019322 | pentose biosynthetic process | 3 | NP_000393, NP_002854, NP_002622 | 2.36×10^–3^ |
| GO:0006572 | tyrosine catabolic process | 3 | NP_000178, NP_000128, NP_002141 | 7.10×10^–3^ |
| GO:0006575 | cellular amino acid derivative metabolic process | 7 | NP_001116105, NP_000393, NP_005887, NP_001076440, NP_002530, NP_000445, NP_000687 | 7.57×10^–3^ |
| GO:0006559 | L-phenylalanine catabolic process | 3 | NP_000178, NP_000128, NP_002141 | 9.95×10^–3^ |
| GO:0046487 | glyoxylate metabolic process | 3 | NP_005887, NP_036335, NP_000021 | 9.95×10^–3^ |

*^a^* The number of proteins from the input list (control, 126; cold exposure, 100) classified by the GO:BP term.

**Table S4. KEGG pathways associating with detected protein groups**

The enriched analysis of Kyoto Encyclopedia of Genes and Genomes (KEGG) pathways was performed using the DAVID program with candidate human homologues. False discovery rate (FDR)-corrected *P* values were defined by modified Fisher’s exact test using the Benjamini and Hochberg FDR correction. The significantly enriched pathways (FDR-corrected *P* < 0.01) are shown with FDR-corrected *P* values.

| **Pathway ID** | **KEGG Pathway** | **Count***^a^* | | **Proteins (RefSeq Accession, human)***^b^* | | **FDR-corrected**  ***P* value** | |
| --- | --- | --- | --- | --- | --- | --- | --- |
|  |  | **CNT** | **CE** | **Control** | **Cold exposure** | **Control** | **Cold exposure** |
| hsa00010 | Glycolysis / Gluconeogenesis | 12 | 9 | NP_002037, NP_001121100, **NP_697021**, NP_000660, NP_001158886, NP_000026, **NP_001419**, NP_001152759, NP_001967, NP_000166, **NP_002645**, NP_005557, NP_000687 | NP_002037, NP_000026, NP_001158886, NP_001152759, NP_001121100, NP_001967, NP_000166, NP_005557, NP_000687, NP_000660 | 1.58×10^–8^ | 1.06×10^–5^ |
| hsa00030 | Pentose phosphate pathway | 8 | 8 | NP_006746, NP_000393, NP_000026, NP_001121100, NP_000166, NP_002622, NP_001128527, NP_115512 | NP_006746, NP_000393, NP_000026, NP_001121100, NP_000166, NP_002622, NP_001128527, NP_115512 | 8.10×10^–7^ | 5.30×10^–7^ |
| hsa00250 | Alanine, aspartate and glutamate metabolism | 7 | 6 | NP_001116105, **NP_002070**, NP_000041, NP_000021, NP_005262, NP_004332, NP_000039 | NP_001116105, NP_000041, NP_000021, NP_005262, NP_004332, NP_000039 | 6.30×10^–5^ | 5.25×10^–4^ |
| hsa00330 | Arginine and proline metabolism | 8 | 7 | NP_001116105, **NP_002070**, NP_000041, NP_002530, NP_000036, NP_005262, NP_000687, NP_000039 | NP_001116105, NP_000041, NP_002530, NP_000036, NP_005262, NP_000687, NP_000039 | 9.66×10^–5^ | 4.67×10^–4^ |
| hsa00620 | Pyruvate metabolism | 7 | 6 | NP_001158886, NP_036335, NP_005882, NP_005557, **NP_002645**, NP_005909, NP_005908, NP_000687 | NP_001158886, NP_036335, NP_005882, NP_005557, NP_005909, NP_005908, NP_000687 | 1.79×10^–4^ | 1.12×10^–3^ |

*^a^* Number of proteins from the input list (control, 126; cold exposure, 100) classified by the pathway ID. Abbreviations are: CNT, control; CE, cold exposure.

*^b^* Proteins that are specific to the control or cold-exposure conditions are in bold.
